# KCTD7 mutations impair the trafficking of lysosomal enzymes through CLN5 accumulation to cause neuronal ceroid lipofuscinoses

**DOI:** 10.1126/sciadv.abm5578

**Published:** 2022-08-03

**Authors:** Yalan Wang, Xiaotong Cao, Pei Liu, Weijia Zeng, Rui Peng, Qing Shi, Kai Feng, Pingzhao Zhang, Huiru Sun, Chenji Wang, Hongyan Wang

**Affiliations:** ^1^ Obstetrics and Gynecology Hospital, NHC Key Laboratory of Reproduction Regulation, Shanghai Institute of Planned Parenthood Research, State Key Laboratory of Genetic Engineering, School of Life Sciences, Children’s Hospital, Fudan University, Shanghai, China.; ^2^ Shanghai Key Laboratory of Metabolic Remodeling and Health, Institute of Metabolism and Integrative Biology, Institute of Reproduction and Development, Fudan University, Shanghai, China.; ^3^ Department of Pathology, School of Basic Medical Sciences, Fudan University Shanghai Cancer Center, Fudan University, Shanghai, China.

## Abstract

Lysosomes are central organelles for cellular degradation and energy metabolism. Neuronal ceroid lipofuscinoses (NCLs) are a group of the most common neurodegenerative lysosomal storage disorders characterized by intracellular accumulation of ceroid in neurons. Mutations in *KCTD7*, a gene encoding an adaptor of the CUL3-RING E3 ubiquitin ligase (CRL3) complex, are categorized as a unique NCL subtype. However, the underlying mechanisms remain elusive. Here, we report various lysosomal and autophagic defects in KCTD7-deficient cells. Mechanistically, the CRL3-KCTD7 complex degrades CLN5, whereas patient-derived KCTD7 mutations disrupt the interaction between KCTD7-CUL3 or KCTD7-CLN5 and ultimately lead to excessive accumulation of CLN5. The accumulated CLN5 disrupts the interaction between CLN6/8 and lysosomal enzymes at the endoplasmic reticulum (ER), subsequently impairing ER-to-Golgi trafficking of lysosomal enzymes. Our findings reveal previously unrecognized roles of KCTD7-mediated CLN5 proteolysis in lysosomal homeostasis and demonstrate that KCTD7 and CLN5 are biochemically linked and function in a common neurodegenerative pathway.

## Teaser

Disruption of KCTD7-regulated CLN5 proteolysis leads to neuronal ceroid lipofuscinoses, type 14.

## INTRODUCTION

Lysosomes, with their uniquely acidic pH and acid hydrolases, are subcellular organelles central to cellular degradation, nutrient sensing, plasma membrane repair, exocytosis, and regulated cell death. The physiological importance of lysosomal homeostasis has been illustrated by the discovery of more than 70 inherited diseases called lysosomal storage disorders (LSDs) (*1*, *2*). LSDs are characterized by lysosomal dysfunction associated with accumulation of nondegraded materials (proteins, lipids, oligosaccharides, etc.) in lysosomes. Neurodegeneration is a prominent manifestation in most LSDs. Neuronal ceroid lipofuscinoses (NCLs), commonly called Batten disease, constitute a subgroup of the most common LSDs. At the cellular level, NCLs show excessive accumulation of autofluorescent lipopigments (called ceroids) in neurons and progressive neuronal loss (*3*–*10*). While the clinical symptoms vary across the NCL subtypes, several common features have been reported, including epileptic seizures, vision loss, progressive motor and cognitive decline, and eventual premature death (*3*, *5*, *6*, *10*–*12*).

NCLs result from mutations in at least 13 CLN genes encoding soluble or transmembrane (TM) proteins localized in endosomes/lysosomes (CLN1/PPT1, CLN2/TPP1, CLN3, CLN5, CLN7/MFSD8, CLN10/CTSD, CLN12/ATP13A2, and CLN13/CTSF), the endoplasmic reticulum (ER) (CLN6 and CLN8), and the cytosol (CLN4/DNAJC5 and CLN14/KCTD7) or secreted into the extracellular space (CLN11/GRN). Except for the autosomal dominant model caused by mutation of DNAJC5, NCLs are generally caused by recessive loss-of-function mutations. Usually, the causative gene mutations in NCLs result in defects in lysosomal enzyme activities or impaired trafficking of lysosomal enzymes to dysregulate lysosomal homeostasis (*3*).

The largest E3 subclass is the Cullin-RING ligases (CRLs), which are modular, multisubunit enzymes comprising hundreds of distinct CRL complexes with the capacity to recruit numerous substrates. The CRL3 complex is composed of Cullin 3 (CUL3) and RING-Box 1 (RBX1) together with a BTB (BR-C, ttk and bab) domain–containing adaptor protein for substrate binding (*13*). KCTD7 is a member of the BTB domain–containing adaptor subfamily denoted KCTD (proteins containing a K^+^ channel tetramerization domain). Most KCTD subfamily members act as substrate-binding receptors for CRL3 (*14*). Patients with KCTD7 mutations were first diagnosed with progressive myoclonic epilepsy type 3 (EPM3) (*15*–*17*). Subsequently, the pathological features of ceroid/lipofuscin in peripheral blood and skin biopsy of patients allowed the inclusion of KCTD7 mutation diseases in a subtype of NCL, and the causative *KCTD7* gene was named as *CLN14* (*18*). A group of patient-derived missense mutations in the BTB domain of *KCTD7* can disrupt the interaction between KCTD7 and CUL3, which may account for a subset of CLN14 cases (*18*). However, more than half of the patient-derived *KCTD7* mutations are located far from the BTB domain, and the mechanisms by which these mutations affect the formation of the CRL3-KCTD7 complex or the recognition of substrates are largely unknown. The physiological substrates and cellular functions of the CRL3-KCTD7 complex have not yet been identified, impeding the establishment of potential therapeutic strategies.

To delineate the molecular mechanisms underlying KCTD7-mutated NCLs, we combined CRISPR-Cas9–mediated gene tagging and affinity purification followed by mass spectrometry (AP-MS) to identify potential KCTD7-interacting partners, especially proteolytic substrates. We found that KCTD7 acts as a CRL3 adaptor to recruit CLN5 for degradation via the ubiquitin-proteasome pathway. KCTD7 deficiency directly leads to impaired trafficking of lysosomal enzymes and, ultimately, lysosomal dysfunction as a result of excessive CLN5 accumulation.

## RESULTS

### Accumulation of lysosomal storage materials and dysregulation of the lysosomal enzyme composition in *KCTD7*-deficient cells

**Fig. 1.**
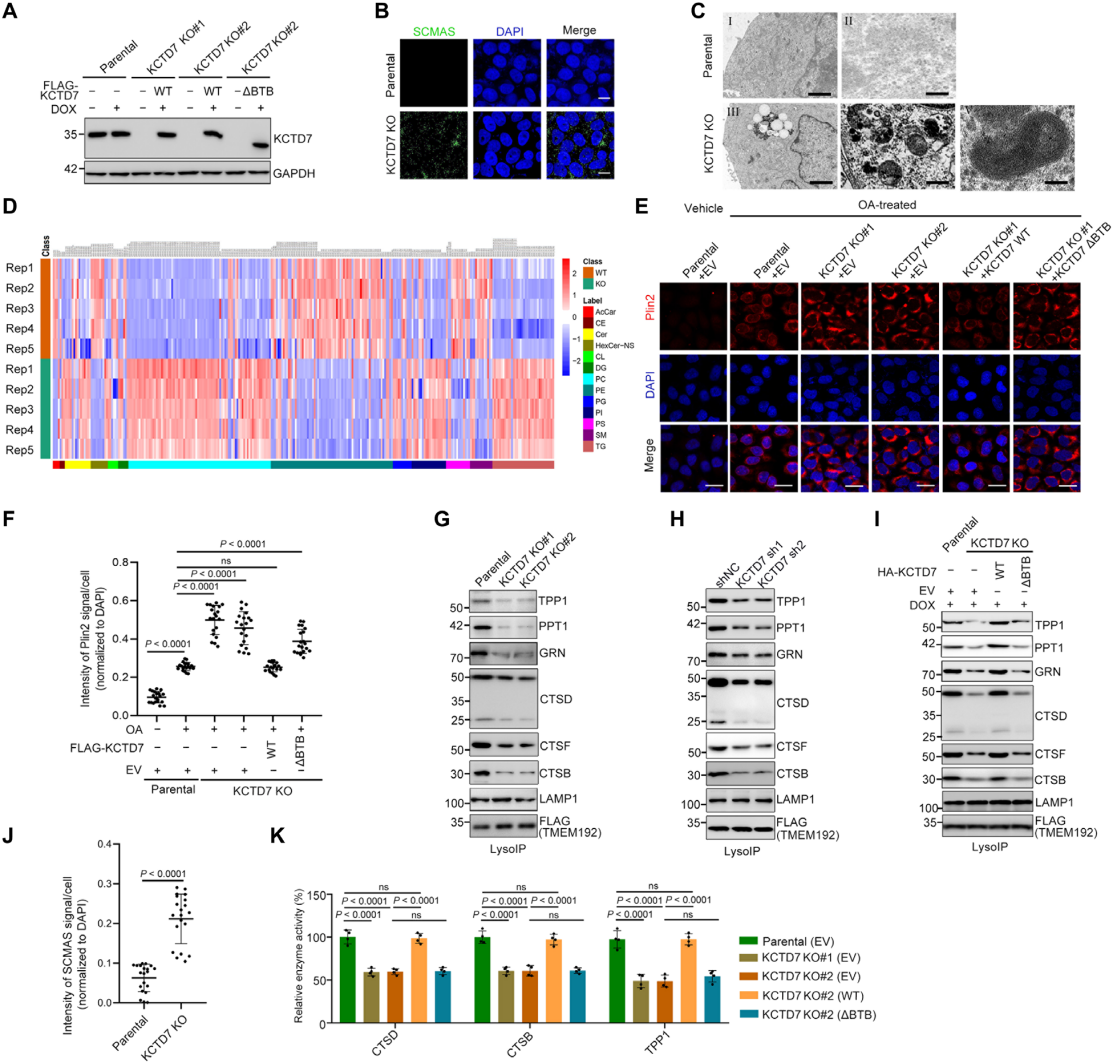
KCTD7 deficiency leads to altered lipid metabolism and lysosomal defects. (**A**) Exogenous KCTD7 was reintroduced into two KCTD7 knockout (KO) cell clones to generate KCTD7 (WT or ΔBTB) Tet-On inducible cells. All cell lines were treated with dimethyl sulfoxide (DMSO) or doxycycline (DOX) (10 ng/ml) for 3 days, and whole-cell lysates (WCLs) were prepared for Western blot (WB) analysis. Parental HeLa cells were used as control. (**B**) Representative IF images of parental and KCTD7-deficient HeLa cells stained with SCMAS and 4′,6-diamidino-2-phenylindole (DAPI). Scale bars, 50 μm. (**C**) TEM images showing lipids (III), GRODs (IV), and fingerprint-like structures (V) in KCTD7-deficient HeLa cells but not in parental cells (I and III). Scale bars, 2 μm (I and III) and 200 nm (II, IV, and V). (**D**) Lipidomic heatmap showing fold changes of lipid species in parental and KCTD7-deficient HeLa cells (*n* = 5). (**E** and **F**) The indicated HeLa cells were treated with vehicle or Bovine serum albumin (BAS)–coupled oleate acid (OA; 400 μM) for 12 hours at 33°C and were then used for IF staining of Plin2 and DAPI (blue) (E). Scale bars, 50 μm. Quantification of the Plin2 fluorescence intensity is shown in (F). Data are presented as means ± SD (*n* = 20). (**G**) Lysosomal fractions from the indicated HeLa cells were isolated using lysosome immunoprecipitation (LysoIP) and were then subjected to WB analysis. (**H** and **I**) Lysosomal fractions from the indicated HeLa cells were isolated using LysoIP and were then subjected to WB analysis. (**J**) Quantification of SCMAS signal intensity per cell is shown in (B). Data are presented as means ± SD (*n* = 20). (**K**) Relative enzymatic activities of CTSD, CTSB, and TPP1 in the indicated HeLa cells. *P* values are calculated using Student’s *t* test (J) and one-way analysis of variance (ANOVA) test (F and K). ns, not statistically significant.

The hallmark pathological characteristic of human NCLs, including KCTD7 mutation–related NCLs, is the accumulation of highly autofluorescent and pigmented intracellular storage deposits (*3*). Several previous studies reported that characteristic ultrastructural features of lipid droplets (LDs), fingerprint-like profiles, and granular osmiophilic deposits (GROD) in patient-derived fibroblasts, neurons, and lymphocytes but other classic NCL features, such as cytosomes, rectilinear profiles, and curvilinear morphologies, were not observed (*17*–*19*). However, whether these key disease-specific phenotypes can be reproduced in cultured cells in vitro is unclear. To address this issue, we successfully ablated *KCTD7* expression by CRISPR-Cas9 gene editing in HeLa cells, which exhibit abundant KCTD7 expression (Fig. 1A and fig. S1, A and B) (*20*). We determined the nature of the storage material by immunofluorescence (IF) for subunit c of mitochondrial adenosine triphosphate synthase (SCMAS), a major protein known to accumulate in CLN14-related lipofuscin (*18*). KCTD7-deficient HeLa cells, but not the parental cells, were strongly immunostained for SCMAS (Fig. 1, B and J). Transmission electron microscopy (TEM) has previously been used to categorize the ultrastructural characteristics of storage material profiles in human NCL biopsies (*21*). Our TEM analysis revealed several NCL-like features in KCTD7-deficient HeLa cells compared to parental cells. LDs, GROD, and fingerprint-like structures were frequently observed in KCTD7-deficient cells, consistent with observations in KCTD7-mutated patient biopsies (Fig. 1C) (*18*). Lipidomic analysis showed that phosphatidylcholine, phosphatidylinositol, triacylglycerol, and ceramide were markedly increased, whereas acylcarnitine, hexosylceramide, phosphatidylethanolamine, glycerophosphoserine, diacylglycerol, and sphingomyelin were markedly decreased in KCTD7-deficient cells (Fig. 1D and data S1). The drastic changes in lipid species induced by KCTD7 deficiency indicate a pivotal role of KCTD7 in maintaining lipid homeostasis, as observed in other NCL models (*22*).

The following rescue experiment results further suggested that the impaired capability for LD degradation was specifically caused by KCTD7 deficiency. A Tet-On inducible expression system was used to achieve exogenous KCTD7 expression in KCTD7-deficient cells at a level comparable to that in parental cells (Fig. 1A). Compared with that in parental cells, the IF intensity of perilipin 2 (Plin2), an LD envelope protein, was substantially increased in KCTD7-deficient cells upon oleate acid treatment. The increase in Plin2 was reversed by reintroduction of Tet-On inducible KCTD7-WT (wild type) (Fig. 1A) but not the ΔBTB mutant (incapable of assembling an active CRL3 complex; Fig. 1, E and F). Similar results were obtained by neutral lipid staining with the dye BODIPY 493/503 (fig. S2, A and B). In addition, we unexpectedly found that glycogen particles were frequently present in KCTD7-deficient cells but not in parental cells, as detected by TEM and further confirmed by the use of a glycogen-specific antibody (fig. S2, C to E). These results suggested the impaired capability for LD and glycogen degradation caused by KCTD7 deficiency.

NCLs are characterized by lysosomal dysfunction. KCTD7-deficient cells showed a morphology similar to that of parental cells, as assessed by staining of the lysosomal marker Lysosomal-associated membrane protein 1 (LAMP1) (fig. S2, F and G). Dysregulation of lysosomal acidification impairs the activities of various acid hydrolases, which suppresses their degradative capabilities and causes accumulation of undegraded cargo in lysosomes. Our results showed that the fluorescence permeability of the pH-dependent LysoSensor probe in KCTD7-deficient cells was substantially lower than that in parental cells (fig. S2, H and I), suggesting an elevated lysosomal pH.

Impaired trafficking of lysosomal enzymes causes lysosomal dysfunction, as previously demonstrated in CLN3, CLN5, CLN6, CLN7, and CLN8 deficiency (*23*–*27*). KCTD7 knockout (KO) or short hairpin RNA (shRNA)–mediated knockdown (KD) markedly decreased the proportions of these enzymes, such as TPP1, PPT1, GRN, CTSD, CTSF, and Cathepsin B (CTSB), in lysosomes but did not alter the corresponding total protein levels in the whole-cell lysates (WCLs) (Fig. 1, G and H). In addition, we observed a decrease in the enzymatic activities of CTSD, CTSB, and TPP1 in KCTD7-deficient cells (Fig. 1K). Furthermore, reintroduction of KCTD7-WT but not the ΔBTB mutant reversed KCTD7 KO-induced alterations in the composition and activities of lysosomal enzymes (Fig. 1, I and K). Similar results were observed in KCTD7 KO U87-MG glioblastoma cells (fig. S3, A to M) and KCTD7 KD SH-SY-5Y neuroblastoma cells (fig. S4, A to J).

Together, our findings suggest that KCTD7 deficiency leads to the accumulation of lysosomal storage materials, reminiscent of observations in KCTD7-mutated NCLs (*18*). KCTD7 deficiency also impairs lysosomal enzyme composition and activity, a common feature of NCLs (*26*, *27*–*30*).

### KCTD7 deficiency leads to autophagic defects, mTORC1 inactivation, unfolded protein response activation, and increased susceptibility to lysosome-dependent cell death

**Fig. 2.**
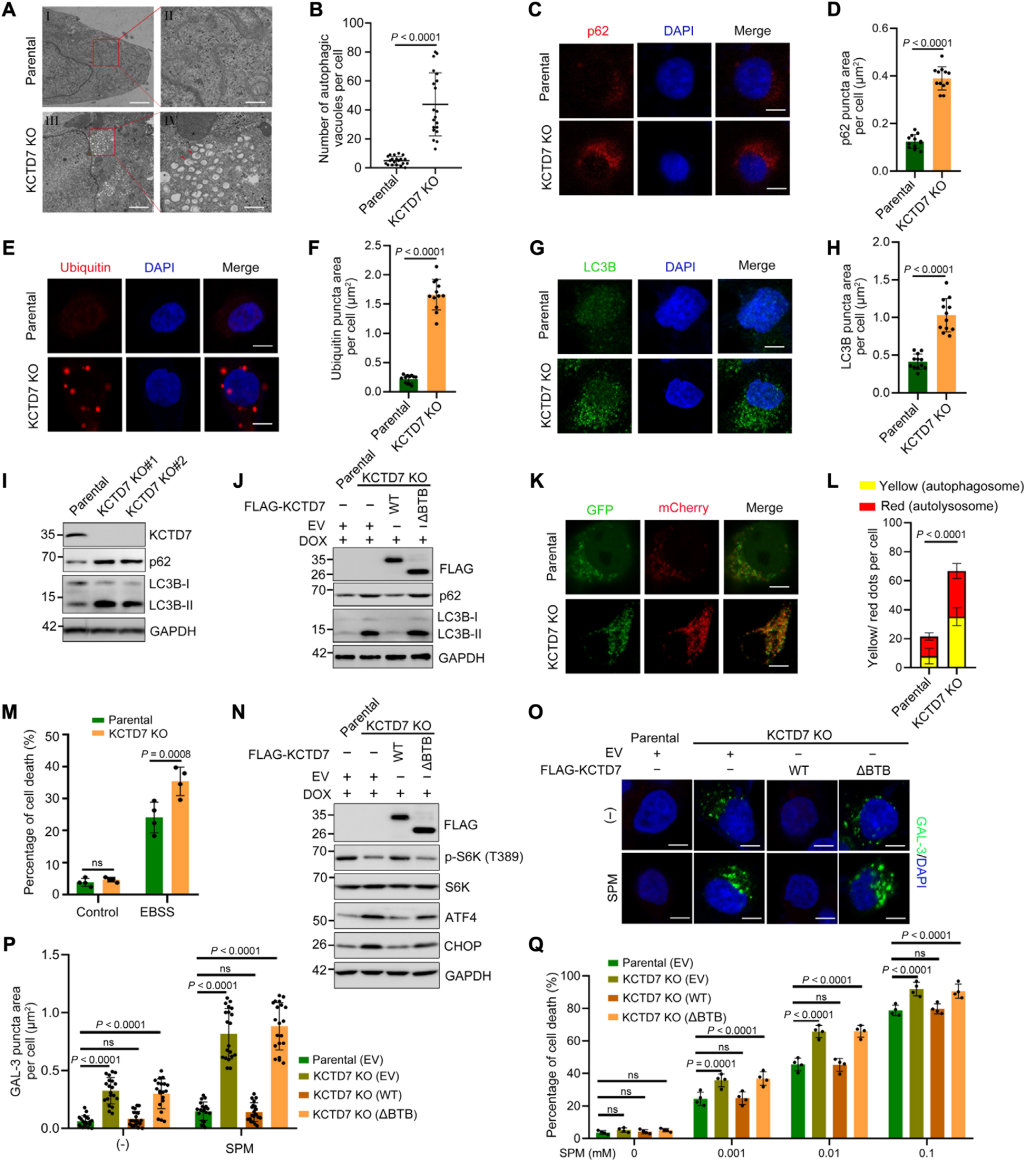
KCTD7 deficiency leads to autophagic defects, UPR activation, and increased susceptibility to lysosome-dependent cell death. (**A** and **B**) TEM images showing the accumulation of autophagic vacuoles (indicated with red arrows) in KCTD7 KO HeLa cells (A). Scale bars, 2 μm (I and III) and 500 nm (II and IV). (**C** to **H**) Representative IF images of the indicated HeLa cells stained with p62 (C), ubiquitin (E), LC3B (G), and DAPI. Scale bars, 10 μm. Quantification of the intensity of p62 (D), ubiquitin (F), and LC3B (H) signals per cell (D). Data are shown as means ± SD (*n* = 12). (**I** and **J**) WB analysis of autophagic markers in WCLs from the indicated HeLa cells. (**K** and **L**) Representative mCherry-GFP-LC3–tandem IF images of the indicated HeLa cells (K). Scale bars, 10 μm. Quantification of the yellow (autophagosome) and red (autolysosome) signal intensity per cell (L). Data are shown as means ± SD (*n* = 12). (**M**) Cell death rate (propidium iodide staining) of parental and KCTD7-deficient HeLa cells incubated in control medium [Dulbecco’s modified Eagle’s medium (DMEM)] or EBSS for 3 hours. (**N**) WB analysis of WCLs from the indicated HeLa cells. (**O** and **P**) Representative IF images of galectin 3 (GAL-3) staining in the indicated HeLa cells. The cells were treated with/without 0.01 mM SPM for 12 hours. Scale bars, 10 μm. Quantification of the area of GAL-3 puncta per cell is shown in (P). Data are presented as means ± SD (*n* = 20). (**Q**) Cell death rate of the indicated HeLa cells upon treatment with increasing concentrations of SPM for 24 hours. Data are presented as means ± SD (*n* = 3). *P* values are calculated using Student’s *t* test (B, D, F, H, and L), one-way ANOVA test (M), and two-way ANOVA test (P and Q).

Another prominent feature of KCTD7-deficient cells is accumulation of late autophagosome-like structures engorged with both electron-dense lysosomes and electron-lucent LDs (Fig. 2, A and B). IF analysis revealed significant increases in the numbers of p62, ubiquitin, and LC3B puncta in *KCTD7*-deficient cells, which were barely detectable in parental cells (Fig. 2, C to H). Western blot (WB) analysis showed that the accumulation of p62 and LC3B-II in KCTD7-deficient cells was reversed by reintroduction of KCTD7-WT but not the ΔBTB mutant (Fig. 2, I and J). The autophagic flux assays using the mCherry-GFP-LC3B reporter revealed that KCTD7 deficiency markedly increased the formation of both autophagosomes and autolysosomes, suggesting defective autophagy (Fig. 2, K and L). We then investigated whether the autophagic defects caused by KCTD7-deficiency affect nutrient deprivation-induced cell death. As shown in Fig. 2M, treatment with Earle’s balanced salt solution (EBSS) led to more pronounced cell death in KCTD7-deficient cells than in parental cells. Together, our data indicate that KCTD7 is required for autophagic homeostasis.

Attenuated mammalian target of rapamycin complex 1 (mTORC1) signaling has been observed in other NCL subtypes, such as CLN3 and CLN7 (*27*, *31*). The phosphorylation level of S6K, an indicator of mTORC1 activity, was markedly decreased in KCTD7-deficient cells and was restored by reintroduction of KCTD7-WT but not the ΔBTB mutant (Fig. 2N). Abnormal activation of the unfolded protein response (UPR) by severe ER stress has been reported in several NCL subtypes (*23*, *26*). We found that the UPR target genes activating transcription factor 4 (ATF4) and C/EBP Homologous Protein (CHOP) were markedly up-regulated in KCTD7-deficient cells, suggesting a persistent UPR activation in the absence of KCTD7 (Fig. 2N).

At high concentrations, spermine can act as a lysosomotropic agent to induce lysosomal membrane rupture and lysosome-dependent cell death (LCD) (*32*). Spermine treatment led to significant increases in the number and size of galectin 3 (GAL-3; a cytosolic galectin frequently used to monitor lysosomal damage) puncta, a marker of lysosomal rupture, in KCTD7-deficient cells compared to parental cells (Fig. 2, O and P). Last, we demonstrated that KCTD7-deficient cells were more susceptible to cell death induced by high concentrations of spermine (Fig. 2Q). Together, our findings suggest that KCTD7 deficiency leads to autophagic defects, impaired mTORC1 activation, persistent UPR activation, and increased susceptibility to LCD.

### Identification of CLN5 as a KCTD7-interacting protein

**Fig. 3.**
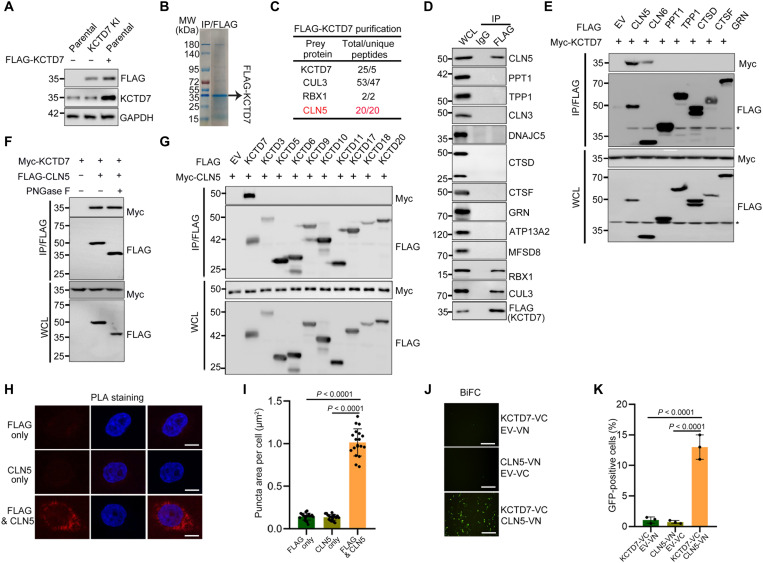
KCTD7 interacts with CLN5 in cells. (**A**) Verification of HeLa cells expressing endogenous FLAG-KCTD7 by WB analysis. GAPDH, glyceraldehyde-3-phosphate dehydrogenase. (**B** and **C**) Tandem affinity purification of KCTD7 protein complex was conducted in FLAG-KCTD7 KI HeLa cells. Associated proteins were separated by SDS–polyacrylamide gel electrophoresis and visualized by Coomassie blue staining (B). MW, molecular weight. The numbers of total/unique peptides identified by MS analysis are shown in (C). (**D**) FLAG-KCTD7 KI HeLa cells were collected and subjected to co-IP with immunoglobulin G (IgG) or an anti-FLAG antibody. WCLs and immunoprecipitates were prepared for WB analysis with the indicated antibodies. (**E**) WB analysis of the indicated proteins in WCLs and anti-FLAG co-IP samples from 293T cells transfected with the indicated plasmids and treated with MG132 (10 μM, 8 hours) before harvesting. (**F**) WB analysis of the indicated proteins in WCLs and anti-FLAG co-IP samples from 293T cells. Cells were transfected with the indicated plasmids, harvested, and then treated with/without PNGase F for 12 hours at 37°C before co-IP. (**G**) WB analysis of the indicated proteins in WCLs and anti-FLAG co-IP samples from 293T cells transfected with the indicated plasmids and treated with MG132 (10 μM, 8 hours) before harvesting. (**H** and **I**) Representative IF images of the KCTD7-CLN5 interaction in HeLa cells using Duolink PLA (H). Single-antibody (anti-FLAG or anti-CLN5) staining is shown as a negative control. Scale bars, 10 μm. (I) Quantification of the PLA signal intensity per cell. Data are presented as means ± SD (*n* = 20). (**J** and **K**) Representative IF images of BiFC assays in HeLa cells transfected with indicated plasmids (J). Scale bars, 200 μm. Percentages of green fluorescent protein (GFP)–positive cells (K). Data are presented as means ± SD (*n* = 3). *P* values are calculated by one-way ANOVA test (I and K).

We performed AP-MS to identify the interacting partners of KCTD7. To minimize nonspecific binding between exogenously overexpressed bait and cellular proteins, a FLAG tag was integrated to the C terminus of the endogenous *KCTD7* locus in HeLa cells using CRISPR-Cas9 gene editing. The presence of KCTD7-FLAG was verified by Sanger sequencing of genomic DNA and by WB analysis (Fig. 3A and fig. S1, C and D). KCTD7 protein complexes were purified and identified by AP-MS. CLN5, in addition to CUL3 and RBX1, was ranked as a high-confidence interactor on the interaction hit list. No other NCL-related proteins were detected (Fig. 3, B and C, and data S3). Given that CLN5 mutations also lead to an NCL subtype, we investigated any possible link between KCTD7 mutation–induced NCLs and the KCTD7-CLN5 interaction. We first confirmed the binding of KCTD7 with CLN5 via coimmunoprecipitation (co-IP) assays in KCTD7-FLAG knock-in (KI) cells. Except for weak binding to CLN6, KCTD7 showed no interaction with any other reported NCL-related protein (Fig. 3, D and E). CLN5 was reported as a glycoprotein. Treatment of cell lysates with recombinant glycosidase [peptide *N*-glycosidase (PNGase) F] to remove high mannose type N-linked glycans resulted in a marked reduction in size from ∼50 to ∼35 kDa, indicating that CLN5 is heavily glycosylated. Co-IP assay results showed that PNGase F treatment–induced CLN5 deglycosylation had no obvious impact on the KCTD7-CLN5 interaction (Fig. 3F). Moreover, we did not observe an interaction of CLN5 with any other KCTD subfamily members examined (Fig. 3G). Notably, the in situ proximity ligation assay (PLA) revealed that the KCTD7-CLN5 interaction occurs in cytoplasmic compartments (Fig. 3, H and I). Last, we performed bimolecular fluorescence complementation (BiFC) assays, which enabled direct visualization of the KCTD7-CLN5 interaction in living cells (Fig. 3, J and K). Together, our findings suggest that KCTD7 specifically interacts with CLN5 in cells.

### KCTD7 promotes proteasomal degradation of CLN5

**Fig. 4.**
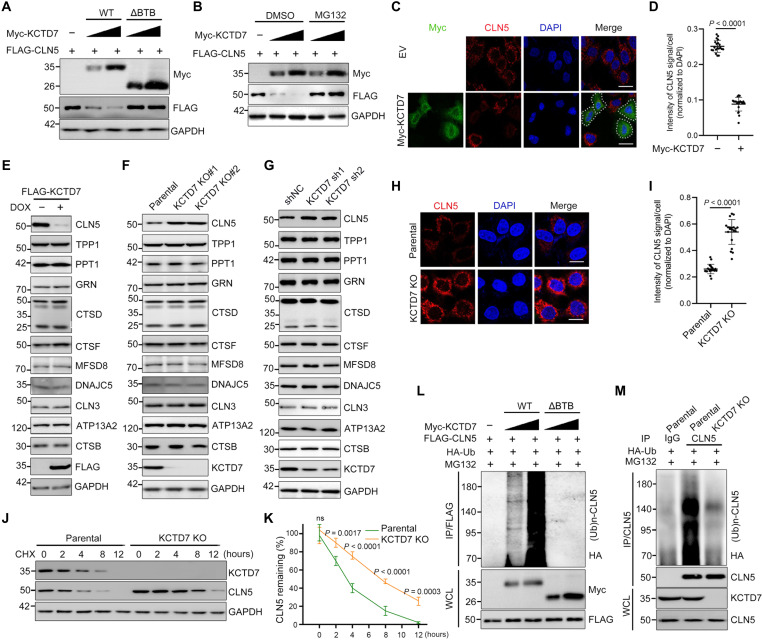
KCTD7 promotes the degradation and ubiquitination of CLN5. (**A**) WB analysis of the indicated proteins in WCLs from 293T cells transfected with the indicated plasmids. (**B**) 293T cells were transfected with the indicated plasmids and were then treated with DMSO or MG132 (10 μM) for 8 hours. WCLs were prepared for WB analysis. (**C** and **D**) Representative images of HeLa cells transfected with the indicated plasmids and stained with an Myc (green), CLN5 (red), and DAPI (blue) (C). Scale bars, 30 μm. (D) Quantification of the CLN5 signal intensity per cell. Data are presented as means ± SD (*n* = 20). (**E**) WB analysis of the indicated proteins in WCLs from KCTD7 Tet-On inducible HeLa cells treated with DMSO or DOX (10 ng/ml) for 24 hours. (**F**) WB analysis of the indicated proteins in WCLs from the indicated HeLa cells. (**G**) WB analysis of the indicated proteins in WCLs from the indicated HeLa cells. (**H** and **I**) Representative IF images of the indicated HeLa cells stained with an anti-CLN5 antibody and DAPI. Scale bars, 30 μm. Quantification of the CLN5 signal intensity per cell (M). Data are presented as means ± SD (*n* = 20). (**J** and **K**) WB analysis of the indicated proteins in WCLs of the indicated HeLa cells treated with cycloheximide (CHX; 50 μg/ml) and harvested at different time points. Data are presented as means ± SD (*n* = 3). (**L**) In vivo ubiquitination assays of 293T cells transfected with the indicated plasmids and treated with MG132 (10 μM) for 8 hours. (**M**) In vivo ubiquitination assays of parental and KCTD7-deficient HeLa cells transfected with the indicated plasmids and treated with MG132 (10 μM) for 8 hours. *P* values are calculated using Student’s *t* test (D and I) and two-way ANOVA test (K).

Although CLN5 has been reported to regulate lysosomal enzyme trafficking, little is known about the mechanism by which CLN5 function is regulated by posttranslational modifications (PTMs). Treatment of HeLa cells with the proteasome inhibitor MG132 increased the CLN5 protein level but not the corresponding mRNA level. MLN4924, a small-molecule inhibitor of CRL complexes, also caused accumulation of CLN5 proteins but not mRNA transcripts (fig. S5, A and B). We depleted RBX1 and each Cullin separately in HeLa cells with small interfering RNAs and found that depletion of only RBX1 or CUL3 led to a marked increase in the CLN5 protein level, suggesting that CLN5 stability is regulated by one or more CRL3 E3 ubiquitin ligases (fig. S5C). Exogenous coexpression of KCTD7-WT but not the ΔBTB mutant or any other KCTD family protein markedly decreased the CLN5 protein level, and this effect was blocked by MG132 treatment (Fig. 4, A and B, and fig. S5D). IF and WB analysis showed that exogenous overexpression of KCTD7 led to a marked decrease in the endogenous CLN5 protein level (Fig. 4, C to E). By contrast, depletion of KCTD7 by KO or KD resulted in a marked increase in the endogenous CLN5 protein level but had no impact on the levels of other NCL-related proteins (Figs. 4, F and G). Similar results were observed in KCTD7 KO U87-MG cells and KCTD7 KD SH-SY5Y cells (figs. S3E and S4B). IF analysis showed that CLN5 staining intensity was markedly increased in KCTD7-deficient cells (Fig. 4, H and I), whereas the *CLN5* mRNA level was not affected by KD, KO, or overexpression of KCTD7 (figs. S3D, S4A, and S5, E to G). Moreover, the half-life of CLN5 was markedly prolonged in KCTD7-deficient cells (Fig. 4, J and K). KCTD7-WT, but not the ΔBTB mutant, promoted the K48-linked polyubiquitinataion of CLN5 (Fig. 4L and fig. S5, H and I). Accordingly, the endogenous ubiquitination level of CLN5 was decreased in KCTD7-deficient cells (Fig. 4M).

The aforementioned results also showed that KCTD7 interacted with CLN6 but to a lesser degree than with CLN5. Unfortunately, a commercial antibody capable of detecting endogenous CLN6 is currently unavailable, and we could not detect whether the CLN6 protein level was changed by KCTD7 deficiency. Alternatively, we overexpressed FLAG-CLN6 and FLAG-CLN5 separately in parental and KCTD7-deficient cells. The FLAG-CLN6 protein level showed no difference between KCTD7-deficient and parental control cells. By contrast, FLAG-CLN5 was significantly stabilized in KCTD7-deficient cells than in parental control cells (fig. S5, J to L). Together, our findings suggest that CLN5 is an authentic proteolytic substrate of the CRL3-KCTD7 complex.

### Patient-derived KCTD7 mutants are defective in promoting proteasomal degradation of CLN5

**Fig. 5.**
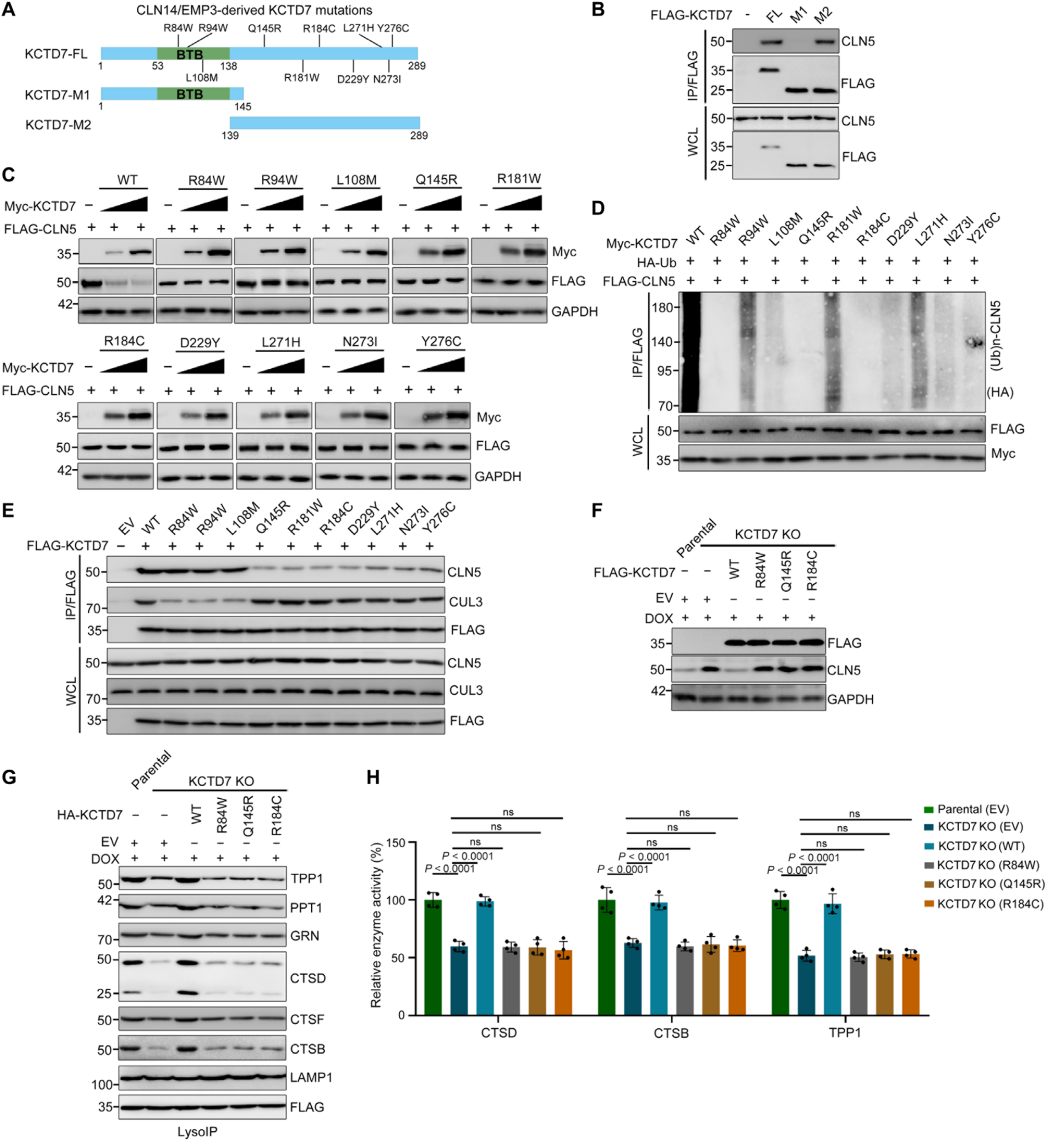
Patient-derived KCTD7 mutants are defective in promoting KCTD7 degradation and fail to reverse lysosomal defects caused by KCTD7 deficiency. (**A**) Distributions of patient-derived KCTD7 mutations and schematic of KCTD7 deletion mutants. (**B**) WB analysis of the indicated proteins in WCLs and anti-FLAG co-IP samples from 293T cells transfected with the indicated plasmids. (**C**) WB analysis of the indicated proteins in WCLs from 293T cells transfected with the indicated plasmids. (**D**) In vivo ubiquitination assays of 293T cells transfected with the indicated plasmids and treated with MG132 (10 μM, 8 hours). The polyubiquitinated forms of CLN5 were detected by WB with the indicated antibodies. (**E**) WB analysis of the indicated proteins in the WCLs and co-IP samples of anti-FLAG antibody obtained from 293T cells transfected with the indicated plasmids and treated with MG132 (10 μM, 8 hours) before harvesting. (**F**) WB analysis of the indicated proteins in WCLs from parental HeLa cells and KCTD7-deficient HeLa cells reintroduced with Empty vector (EV), KCTD7-WT, or KCTD7 mutants. All cell lines were treated with DMSO or DOX (10 ng/ml) for 3 days. (**G**) WB analysis of the indicated proteins from LysoIP of parental HeLa cells and KCTD7-deficient HeLa cells reintroduced with EV, KCTD7-WT, or KCTD7 mutants. All cell lines were treated with DMSO or DOX (10 ng/ml) for 3 days. (**H**) Relative enzymatic activities of CTSD, CTSB, and TPP1 in parental HeLa cells and KCTD7-deficient HeLa cells reintroduced with EV, KCTD7-WT, or KCTD7 mutants. *P* values are calculated by one-way ANOVA test (H).

More than 30 KCTD7 mutations from 37 published patients were located in or outside the BTB domain (table S1). We found that the region between amino acids 139 and 289 contains the majority of mutations and mediates CLN5 binding (Fig. 5, A and B). We postulated that patient-derived KCTD7 mutants may be defective in mediating CLN5 degradation. KCTD7-mediated degradation and ubiquitination of CLN5 were abolished or markedly attenuated in these mutants (Fig. 5, C and D). Consistent with a previous report, KCTD7 mutants with mutations in the BTB domain showed reduced CUL3-binding ability, and we also found that KCTD7 mutants with mutations in the region between amino acids 139 and 289 showed reduced CLN5-binding ability (Fig. 5E and fig. S6A). In contrast to reintroduction of KCTD7-WT, reintroduction of the patient-derived KCTD7 mutants cannot reverse the KCTD7 deficiency–induced CLN5 accumulation (Fig. 5F), reduction in lysosomal enzyme composition and activities (Fig. 5, G and H), and autophagic defects (figs. S6, B and C, and S7, A to H). Together, our findings suggest that the ability of patient-derived KCTD7 mutants to promote CLN5 degradation is severely compromised, resulting in CLN5 accumulation in cells.

### CLN5 deficiency or overexpression leads to lysosomal defects

At first glance, it seems counterintuitive that both CLN5 accumulation induced by KCTD7 deficiency and CLN5 loss-of-function mutations independently lead to NCL subtypes. If CLN5 accumulation induced by either KCTD7 deficiency or CLN5 loss-of-function mutations causes diseases, then CLN5 overexpression could be expected to elicit similar NCL phenotypes. LDs and fingerprint-like structures were frequently observed in HeLa cells stably overexpressing CLN5 (fig. S8A). CLN5 overexpression also led to a reduction in lysosomal enzyme composition and activities and autophagic defects (fig. S8, B to E). As expected, CLN5 KD, which mimics loss-of-function mutations of CLN5, led to a reduction in lysosomal enzyme composition and enzyme activities (fig. S8, F to H). The lipidomic analysis showed that CLN5 KD elicited drastic changes in lipid species, which is largely similar as seen in KCTD7-deficient cells (fig. S8I and data S2). We also found that KCTD7 overexpression, which mimics CLN5 KD, elicited similar NCL-related phenotypes (fig. S9, A to O).

We depleted CLN5 in KCTD7-deficient cells with shRNAs and observed that CLN5 expression was reversed to levels similar to those in the parental cells. Moreover, CLN5 KD largely reversed the KCTD7 deficiency–induced reductions in lysosomal enzyme composition and activities (fig. S10, A to C) and autophagic defects (fig. S10, D to N). Last, we found that KCTD7/CLN5 double KO cells elicited similar NCL phenotypes as KCTD7 KO cells (fig. S11, A to F), which indicated that as a substrate of KCTD7, the dosage balance of CLN5 works vitally in the lysosomal enzyme trafficking. Together, our findings suggest that KCTD7 deficiency–induced lysosomal defects are caused by CLN5 accumulation. Moreover, loss-of-function mutations of CLN5 caused similar results (fig. S8, F to H) (*33*).

### KCTD7 deficiency impairs ER-to-Golgi trafficking of lysosomal enzymes

**Fig. 6.**
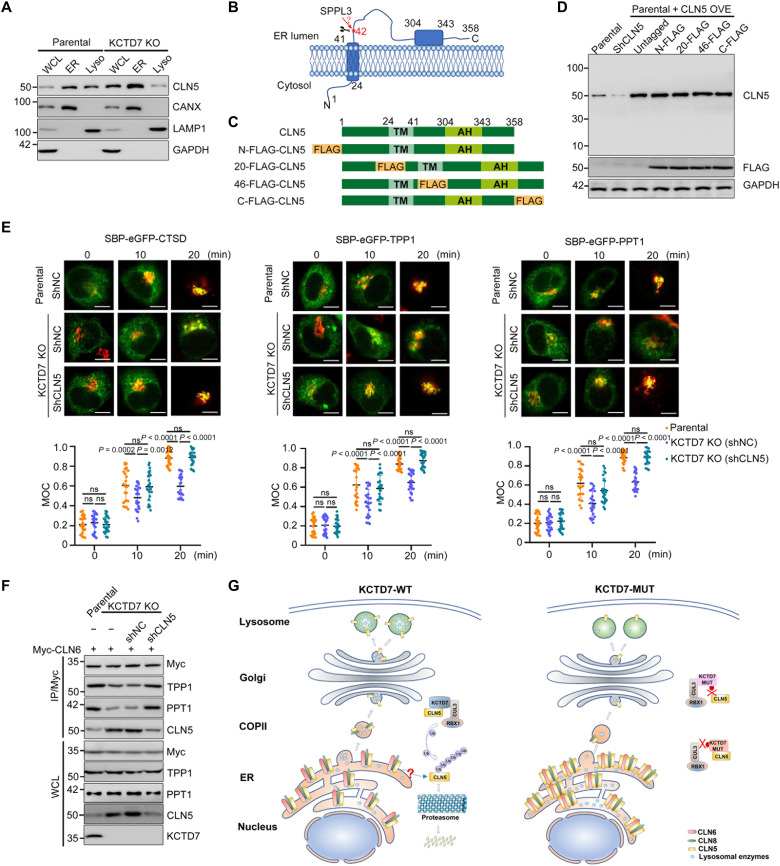
KCTD7 deficiency disrupts lysosomal enzyme trafficking from ER to Golgi through CLN5 accumulation. (**A**) WB analysis of the indicated proteins in the WCLs, ER, and lysosome fractions from parental and KCTD7-deficient HeLa cells. (**B**) Schematic diagram of CLN5 topology in the ER membrane. (**C**) Schematic showing the constructs of CLN5 proteins tagged by N-terminal, internal, or C-terminal FLAG. (**D**) WB analysis of the indicated proteins in WCLs from 293T cells transfected with the indicated plasmids. (**E**) Confocal colocalization assay of SBP-GFP–fused lysosomal enzymes (CTSD, TPP1, and PPT1; green) with the Golgi marker GM130 (red) in parental and KCTD7-deficient HeLa cells stably overexpressing control shRNA or shRNA-targeting CLN5 by retention using selective hook. The SBP-GFP–fused lysosomal enzymes were synchronized at the ER with streptavidin-KDEL (0 min) and competitively released by the addition of d-biotin (10, 20 min) for trafficking. Top: Representative IF images (scale bars, 10 μm). Bottom: Colocalization degree between the test protein and GM130 was quantified by Manders’ overlap coefficient (MOC). Data are presented as means ± SD (*n* = 20). (**F**) Parental and KCTD7-deficient HeLa cells with/without CLN5 KD were transfected with the indicated plasmids for 24 hours. Then, WCLs were prepared and subjected to IP with an anti-Myc antibody. WCLs and immunoprecipitates were prepared for WB analysis with the indicated antibodies. (**G**) Schematic diagram depicting a model in which KCTD7 promotes lysosomal enzyme trafficking and maintains lysosomal function by acting as an endogenous balancer of CLN5. *P* values are calculated using two-way ANOVA test (E).

CLN5 is generally recognized as a lysosomal protein but has also been reported to be localized in the ER, Golgi, and endo/lysosomes (*34*, *35*). Our subcellular fractionation results showed that CLN5 is present in both the ER and lysosomes. In KCTD7-deficient HeLa cells, the protein level of CLN5 in the lysosomal fraction was markedly decreased, although the total level of CLN5 in the WCL markedly increased (Fig. 6A). We proposed that the abnormally accumulated CLN5 may impair the trafficking of lysosomal enzymes from the ER to the Golgi based on the finding that KCTD7 deficiency induced CLN5 accumulation and retention in the ER.

To test this hypothesis, we used a synchronization system called retention using selective hook (RUSH) to monitor ER-to-Golgi trafficking of lysosomal enzymes. In the RUSH system, the test protein is fused to streptavidin-binding protein (SBP) and a green fluorescent protein (GFP) reporter. Second, streptavidin is fused to a KDEL sequence that serves as the ER-targeted hook. At steady state, streptavidin-KDEL is bound to SBP, and the test protein is retained in the ER. Upon biotin addition, the GFP-tagged test protein is released from streptavidin-KDEL (*36*). The subsequent trafficking can be visualized using confocal microscopy. This system was successfully applied in a study demonstrating the role of the CLN6-CLN8 complex in recruiting lysosomal enzymes at the ER for Golgi transfer (*26*). In these assays, internal ribosome entry site–dependent coexpression of streptavidin-KDEL and SBP-GFP–fused CTSD (or TPP1 or PPT1) was achieved in parental and KCTD7-deficient cells. The subcellular localization of CTSD, TPP1, or PPT1 was then monitored over a time course upon biotin-induced synchronous release. The overlap of the enzyme signal with that of the Golgi marker GM130 was quantitatively measured at each time point. As shown in Fig. 6E, KCTD7 deficiency led to a significant delay in ER-to-Golgi trafficking of lysosomal enzymes, which was largely reversed by CLN5 KD. Moreover, ER-to-Golgi trafficking of lysosomal enzymes was delayed in cells with CLN5 KD or overexpression (fig. S12, A and B), suggesting that proper ER-to-Golgi trafficking is sensitive to the CLN5 protein level.

CLN5 is translated as a type II TM protein with a cytoplasmic N terminus, one TM segment, and a large luminal C-terminal domain containing an amphipathic helix (*37*). Whether CLN5 acts mainly as a TM protein or a soluble protein is controversial (*38*–*40*). CLN5 is reported to be cleaved by Signal peptide peptidase-like 3 (SPPL3), a member of the SPP/SPPL intramembrane protease family, into a mature soluble protein (amino acids 50 to 358; Fig. 6B) (*40*). The antibody that we used recognizes the region between amino acids 150 and 250 in CLN5. In contrast to a previous report (*41*), our whole-membrane WB analysis showed only a single CLN5 band near 50 kDa in multiple human cell lines (A498, 22Rv1, SiHa, DU145, HeLa, ECC-1, HEC-1A, SH-SY-5Y, A549, and human embryonic kidney 293T) (fig. S13A). To further clarify whether CLN5 is cleaved in cells, we generated four FLAG-tagged CLN5 constructs, in which a FLAG tag was fused to the N-terminal, C-terminal, or internal region (upstream or downstream of the TM segment) of CLN5 (Fig. 6C). Untagged CLN5 and the FLAG-tagged CLN5 constructs were expressed separately in cells. As shown in Fig. 6D, these proteins were expressed at similar levels, and their molecular weights were the same as those of the endogenous protein. In addition, in analysis of the MS data for C-FLAG–tagged CLN5 purified from cells, we noted multiple peptides corresponding to the N terminus of CLN5 (data S4). In accordance with previous studies, we detected a proportion of secreted CLN5 (approximately 16.7% of the total) in the culture media, and its molecular weight is identical to that of intracellular CLN5 (fig. S13B) (*38*, *42*). Together, our results did not support the conclusion that CLN5 is cleaved to generate a soluble protein in cells (*33*, *36*, *40*).

Since CLN5 was first isolated in 1998, its processing, solubility, and localization have remained controversial (*43*). Because of the lack of antibodies to detect endogenous protein expression, most previous studies were performed using CLN5 overexpression (*29*, *42*, *44*). However, we noted that most of previous studies used *CLN5* transcripts encoding a 407–amino acid protein that is not currently listed in the RefSeq database (*29*, *43*, *44*). Compared to the curated *CLN5* transcript (NM_006493) encoding a 358–amino acid protein, the widely used transcript has an extra 49 amino acids at the N terminus. When a FLAG tag sequence was individually added to the C terminus of the transcripts encoding the 407– and 358–amino acid proteins, the two construct–expressed proteins showed an identical molecular mass (50 kDa), suggesting that the same translation start codon is used by both transcripts. By contrast, the addition of a FLAG tag sequence to the N terminus of the transcript encoding the 407–amino acid protein led to the expression of a protein with a higher molecular weight (60 kDa). Therefore, the addition of an N-terminal tag to the transcript encoding the 407–amino acid protein forces the expression of a protein with a non-naturally occurring 49–amino acid sequence (fig. S13, C and D), which may lead to misinterpretation of the overexpression results in previous studies (*40*, *42*).

Two ER-resident TM proteins, CLN6 and CLN8, form a complex called the ER-to-Golgi relaying of enzymes of the lysosomal system (EGRESS) complex because of its role in recruiting lysosomal enzymes for trafficking to the Golgi (*26*). CLN5 has been reported to interact with CLN6/8 and lysosomal enzymes, but the consequences of these interactions remain elusive (*25*). On the basis of the aforementioned results, we hypothesized that excessive TM CLN5 may compromise CLN6/8 binding with lysosomal enzymes. The interactions between CLN6/8 and lysosomal enzymes (TPP1 or PPT1) were markedly reduced in cells with CLN5 KD or overexpression (fig. S13, E to H), indicating that these interactions can be blocked by either excessive or insufficient amounts of CLN5. The interactions between CLN6/8 and lysosomal enzymes were markedly reduced in KCTD7-deficient cells, and this effect was largely reversed by CLN5 KD (Fig. 6, E and F, and fig. S13, I to K). Together, our findings suggest that the abnormal accumulation of CLN5 induced by KCTD7 deficiency or CLN5 deficiency disrupts CLN6/8-mediated recruitment of lysosomal enzymes, thus impairing ER-to-Golgi transfer of lysosomal enzymes.

## DISCUSSION

The biological functions of KCTD7, the gene mutated in the two rare genetic diseases, CLN14 and EPM3, remain elusive. Few interaction partners of KCTD7 have been identified, except for CUL3 and RBX1 (*13*, *18*). Very little is known about the signaling pathways and biological processes that require intact KCTD7 function. Therefore, therapeutic interventions for KCTD7 mutation–induced fatal disorders are still lacking. By contrast, other NCL-related proteins have been extensively studied and act as lysosomal enzymes or proteins involved in the sorting and trafficking of lysosomal enzymes from the ER to their final destination: lysosomes. We assumed that KCTD7 probably participates in the same pathway as other NCL-related proteins. In the present study, in vitro cellular models of KCTD7 deficiency were generated and successfully reproduced the key pathological features in patients with KCTD7 mutation. In addition, abnormal glycogen deposits were frequently observed in KCTD7-deficient cells. Given that KCTD7 deficiency leads to general autophagic defects, glycophagy may also be impaired. Thus, in-depth histopathological characterization of KCTD7-mutated patient biopsies is warranted to determine whether glycogen deposits were present. We demonstrated that the CRL3-KCTD7 complex mediates proteolytic degradation of CLN5. Patient-derived KCTD7 mutations lead to impairment of either the CUL3-binding or CLN5-binding ability, which allows CLN5 to evade CRL3-KCTD7 complex-mediated degradation. We further demonstrated that KCTD7 deficiency induces CLN5 accumulation and retention at the ER. Excessive CLN5 abrogates EGRESS complex–mediated recruitment of newly synthesized lysosomal enzymes at the ER for subsequent Golgi transfer. Our findings that the aberrant cell phenotypes caused by KCTD7 deficiency were largely rescued by reducing the CLN5 protein level and that CLN5 overexpression phenocopied KCTD7 deficiency in cells suggested that KCTD7 affects a common trafficking pathway by modulating the CLN5 protein level (Fig. 6G).

CLN5 is generally recognized as a soluble lysosomal protein after cleavage of the N-terminal signal peptide for maturation (*40*). A previous report suggested that CLN5 functions as a glycoside hydrolase in lysosomes, but its endogenous substrates have not been identified (*45*). However, CLN5 interacts with NCL-related lysosomal enzymes (CLN1 and 2) and TM proteins (CLN3, -6, -7, and -8) (*25*), strongly suggesting its role beyond a soluble lysosomal protein. We hypothesized that CLN5 is involved in EGRESS complex functions through direct interaction with CLN6/8, probably at the ER membrane. Given that the composition, stoichiometry, and interactions of protein complexes are critical determinants of their biological function, it is expected that both overexpression and KD of CLN5 lead to similar phenotypes. In contrast to CLN6, which is ER-resident, and CLN8, which is ER-resident but also loaded onto COPII vesicles (*26*, *46*), CLN5 has been reported to be localized in the ER, the Golgi, endosome/lysosome (*35*). The anterograde trafficking (Golgi to endosome/lysosome) of soluble lysosomal enzymes is sorted by the lysosomal sorting receptors, such as cation-dependent mannose-6-phosphate receptor (CD-MPR), cation-independent mannose 6-phosphate receptor (CI-MPR), and sortilin (*47*). The efficient retrograde trafficking (endosome to Golgi) of the lysosomal sorting receptors requires the retromer protein complex (*48*). Previous studies demonstrated that CLN5 plays important roles in retrograde trafficking by controlling the localization and activation of the retromer-interacting GTPase Rab7 on the endosomal membrane. Loss of CLN5 led to an impaired recycling of the lysosomal sorting receptors from endosome to trans-Golgi network (*29*, *49*). KCTD7 mutation–induced CLN5 accumulation may also interfere with the retrograde trafficking, thereby reducing the correct sorting of lysosomal enzymes. Further studies are needed to delineate whether dysfunctional KCTD7-CLN5 regulatory axis participates in other steps of protein sorting and trafficking, including anterograde Golgi-to-endosome and retrograde endosome-to-Golgi trafficking.

Previous studies showed that patient-derived CLN5 mutants, such as R121P, R121H, R145P, L358AfsX4, W379C, and Y392X, and the Alzheimer’s disease-linked loss-of-function CLN5 mutant N320S, are retained at the ER rather than trafficked to lysosomes (*42*, *44*, *50*, *51*). The engineered mutations that abolished *N*-glycosylation of CLN5 at N130, -203, -255, or -271 also led to its ER retention (*42*). In CLN5-deficient patient-derived fibroblasts, overexpression of a trafficking-deficient variant of CLN5 that was retained at the ER restricted PPT1 to the ER. In addition, this mutant led to partial retention of CLN3 at the ER (*25*). These findings, together with ours, suggest that the abnormal ER retention of CLN5 caused by proteolytic defects, gene mutations, or aberrant PTMs abrogates the efficient ER-to-Golgi trafficking of its interaction partners and itself.

All NCLs show intracellular accumulation of ceroid deposits and progressive neuronal loss. Although ceroid deposits were originally thought to drive disease progression, it is clear that their accumulation is not a direct cause of neuronal cell death (*52*). Our study suggests that at least three types of cell death may contribute to the neuronal cell death in patients with KCTD7 mutation, as described below. (i) ER stress–induced apoptosis. A common feature of many neurodegenerative disorders is accumulation and deposition of misfolded and unfolded proteins, which triggers ER stress, affects neuronal homeostasis, and leads to cell death (*53*). Our results showed that two UPR targets, ATF4 and CHOP, were markedly up-regulated in KCTD7-deficient cells, suggesting persistent activation of ER stress. (ii) Autophagy. Dysregulation of autophagy has been suggested as a common mechanism in the pathogenesis of NCLs (*54*). We found that KCTD7-deficient cells display autophagic defects and are sensitive to nutrient deprivation–induced cell death. These observations echoed those in a previous report of autophagic defects in fibroblasts from patients with KCTD7 mutation (*17*). (iii) LCD. KCTD7-deficient cells show increased lysosomal membrane rupture, as indicated by GAL-3 release from lysosomes into the cytosol, and are susceptible to spermine-induced LCD (*32*). However, the relative contributions of these three types of KCTD7 mutation–induced cell death to neuronal loss warrant further investigation.

Currently, there is no curative treatment for any NCLs. Considering the previous reports that some NCLs are attributed to CLN5 mutations collectively with our new finding of CLN5 overexpression in KCTD7-mutated NCLs, functional control of CLN5 in balance might be an important target for effective NCL treatment.

## MATERIALS AND METHODS

### Cell lines, cell culture, transfection, and infection

293T, HeLa, U87-MG, SH-SY5Y, A498, 22Rv1, SiHa, DU145, ECC-1, HEC-1A, and A549 cells were obtained from the American Type Culture Collection (ATCC). 293T, HeLa, and U87-MG cells were maintained in Dulbecco’s modified Eagle’s medium (DMEM) supplemented with 10% (v/v) fetal bovine serum (FBS). SH-SY5Y cells were maintained in a 1:1 ratio of ATCC-formulated Eagle’s minimum essential medium (EMEM) and F12 medium supplemented with 10% (v/v) FBS. A498, DU145, and SiHa cells were maintained in EMEM medium supplemented with 10% (v/v) FBS. 22Rv1 and ECC-1 cells were maintained in RPMI 1640 medium supplemented with 10% (v/v) FBS. HEC-1A cells were maintained in ATCC-formulated McCoy’s 5a medium supplemented with 10% (v/v) FBS. A549 cells were maintained in F-12 K medium supplemented with 10% (v/v) FBS. Lipofectamine 3000 was used for transient transfection.

ShRNA-based lentiviral vectors for KCTD7 and CLN5 were purchased from GeneCopoeia. Lentiviruses overexpressing KCTD7 (WT or mutants) or CLN5 were packaged by cotransfection of coding sequence (CDS)–containing backbone plasmids with packaging plasmids into 293T cells, and the viral particles were collected 48 hours after transfection. HeLa and SH-SY5Y cells were infected with these lentiviruses in the presence of polybrene (8 μg/ml) and selected with puromycin (1.5 μg/ml). The sequences of the gene-specific shRNAs are listed in table S2.

### Antibodies and chemicals

Information regarding the antibodies and chemicals used is listed in tables S3 and S4.

### Gene KO/KI cell generation

KO were carried out with the epiCRISPR system as previously reported (*55*). Single-guide RNAs (sgRNAs) targeting human *KCTD7* or *CLN5* were subcloned into the epiCRISPR vector. HeLa and U87 cells were transfected with these constructs. Twenty-four hours after transfection, cells were screened with puromycin (1 μg/ml) for 1 week, and single cells were then isolated in 96-well plates by fluorescence-activated cell sorting (FACS). Ten days later, KO cells were screened by Sanger sequencing and validated by WB analysis. KI of endogenous FLAG-tagged *KCTD7* in HeLa cells was performed as previously reported (*56*). SgRNAs were cloned into the epiCRISPR vector, while donor sequences of the FLAG-tagged CDS flanked by homology arms were cloned into the pUC57 vector. The two constructs were cotransfected into HeLa cells at a 1:1 molar ratio. Then, cells were selected with puromycin and isolated by FACS. KI HeLa cells positive for the FLAG tag were screened by Sanger sequencing and validated by WB analysis. The sequences of the sgRNA are listed in table S6.

### Doxycycline inducible expression

To generate stable cell lines with inducible gene expression, HeLa cells were infected with lentiviruses carrying pGLV-FLAG-KCTD7 (WT or mutants) in the presence of polybrene (8 μg/ml). Stable cell lines were selected in DMEM containing puromycin (1.5 μg/ml) for 3 days. The positive clones were pooled and amplified. Doxycycline (10 ng/ml) was added to stable cells for FLAG-KCTD7 induction.

### RT-qPCR

Total RNA was extracted from parental and KCTD7 KO/KD cells using TRIzol reagent and was then reverse transcribed into cDNA using a HiScript III first Strand cDNA Synthesis Kit (Vazyme). Then, cDNA was amplified using ChamQ SYBR qPCR Master Mix (Vazyme). The primer sequences are listed in table S6. The relative mRNA levels of *KCTD7* and *CLN5* were quantified using the 2^-ΔΔCt^ method with normalization to *GAPDH*.

### Western blot quantification

The WB detection for each experiment was performed in triplicate, and the band intensity of each was quantified by ImageJ according to the manufacturer’s instructions. The quantitative data are shown in figs. S14 to S16.

### Lipidomic analysis

Five replicates of parental and KCTD7 KO or CLN5 KD HeLa cells were harvested for ultrahigh-performance liquid chromatography–high-resolution electrospray ionization tandem MS–based nontargeted lipidomic analysis. Each sample was mixed with internal standards [PC (5 μg/ml) 15:0 to 18:1(d7), PE 15:0 to 18:1(d7), PG 15:0 to 18:1(d7), TAG 15:0 to 18:1(d7)-15:0; lysophosphatidylcholine (2 μg/ml) 18:1(d7); and lysophosphatidylethanolamine 18:1(d7)] and methyl tert-butyl ether (MTBE), and subjected to ultrasonication, centrifugation, and air drying. The samples were suspended in dichloromethane/methanol (1:1, v/v) for lipidomic analysis with a Q Exactive Hybrid Quadrupole Orbitrap MS (Thermo Fisher Scientific) in heated electrospray ionization–positive (HESI+) and –negative (HESI−) modes. The mass/charge ratio of the precursor and product ions was matched against the LipidBlast database. Values are presented as fold changes relative to parental cells. Metabolites with variable importance in projection values larger than 1 in the orthogonal partial least squares discriminant analysis model and *P* < 0.05 were identified as potential differential metabolites and are listed in tables S1 and S2.

### Transmission electron microscopy

Parental and KCTD7 KO or control and CLN5 KD HeLa cells were collected with trypsin-EDTA and washed with phosphate-buffered saline (PBS), followed by fixation with 2.5% glutaraldehyde at room temperature for 2 hours. After dehydration with gradient ethanol and acetone, the samples were sequentially embedded in a 1:1 ratio of acetone:EMBed 812 for 2 to 4 hours at 37°C, a 1:2 ratio of acetone:EMBed 812 overnight at 37°C, and pure EMBed 812 for 5 to 8 hours at 37°C. The samples were placed in a 65°C oven for polymerization for more than 48 hours. Then, the sample blocks were sliced into 60-nm sections with an ultramicrotome for staining. After drying overnight at room temperature, the samples were imaged using a TEM (Talos L120C, Thermo Fisher Scientific).

### IF and confocal microscopy

For IF, HeLa cells were seeded on glass coverslips in 24-well plates and harvested at 80% confluence. The cells were washed with PBS and fixed with 4% paraformaldehyde (PFA) in PBS. After permeabilization with 0.1% Triton X-100, the cells were blocked with 1% bovine serum albumin (BSA). The cells were then incubated with primary antibodies at 4°C overnight and then with secondary antibodies. 4′,6-Diamidino-2-phenylindole (DAPI) was used to stain nuclei. The glass coverslips were mounted on slides and imaged using a confocal microscope (LSM880, Zeiss). Quantitative analyses of proteins were performed using ImageJ software. For Plin2 staining, cells were pretreated with vehicle or BSA-coupled oleic acid at a concentration of 400 μM overnight before collection. The cells were then processed as described above. For BODIPY lipid probe staining, cells were mixed with BODIPY 493/503 and DAPI for 15 min, washed with PBS, and imaged using a confocal microscope.

### Lysosomal enzyme activity assays

Enzyme activities were measured using fluorophore analogs of enzyme substrates as previously described: PPT1 (*57*), CTSD (*58*), and CTSB (*59*).

### Lysosome immunoprecipitation–based lysosome isolation and ER fractionation

HeLa cells transfected with TMEM192-FLAG were used for each lysosome immunoprecipitation as previously reported (*60*). After quick rinsing with PBS, the cells were resuspended in KPBS [136 mM KCl and 10 mM KH_2_PO_4_ (pH 7.25 adjusted with KOH)]. Subsequently, the cells were gently homogenized with 20 strokes using a 2-ml homogenizer and centrifuged at 1000*g* at 4°C for 2 min. The supernatant containing organelles, including lysosomes, was incubated with anti-FLAG antibody-conjugated M2 agarose beads (Sigma-Aldrich) at 4°C for 5 min to isolate the lysosomes with TMEM192-FLAG localized at the membranes. The beads were washed five times with KPBS. The same volume of 2× SDS loading buffer was added to release lysosomal proteins, which were subjected to WB analysis.

ER isolation was carried out using a Minute ER Enrichment Kit (Invent Biotechnologies) according to the manufacturer’s instructions. Briefly, 3 × 10^7^ cells were harvested and washed with cold PBS. The cell pellets were frozen at −80°C for 10 min and then suspended in buffer A. After vigorous vortexing, the suspensions were transferred to a filter cartridge and centrifuged twice as indicated. The supernatants were transferred into fresh tubes and centrifuged. Subsequently, these supernatants were transferred to fresh tubes, buffer B was added, and the tubes were centrifuged. The pellets were resuspended in cold buffer A and vortexed vigorously, and buffer C was then added and vortexed briefly. The mixtures were incubated at room temperature for 15 min. After centrifugation, the supernatants were transferred to fresh tubes, buffer D was added, and the tubes were centrifuged. Last, the pellets were dissolved in 1× SDS loading buffer and subjected to WB analysis.

### Autophagic flux assays

HeLa cells stably overexpressing mCherry-GFP-LC3B were harvested at 80% confluence and imaged using a confocal microscope (LSM880, Zeiss). GFP is vulnerable to the acidic environment of lysosomes and is easily quenched after autophagosomes fuse with lysosomes, whereas mCherry is stable. The yellow dots visible in the merged GFP and mCherry indicate autophagosomes, and the red dots indicate autolysosomes. Quantitative analysis of the yellow and red dots was performed using ImageJ software.

### Cell death assays

For cell death assays, cells were plated into six-well culture dishes before incubation in EBSS for 3 hours (or complete medium as a control). Increasing concentrations of spermine (SPM) were added to the cells for 24 hours. Then, the cells were washed with PBS and fixed with 70% ethanol. These cells were washed again with PBS, stained with propidium iodide and analyzed by flow cytometry to determine the sub-G1 peak.

### LysoSensor staining for lysosome pH measurement

HeLa cells were seeded in glass-bottom dishes and harvested at 80% confluence. Then, the cells were incubated with DMEM containing 1 μM LysoSensor (Yeasen) for 30 min under standard culture conditions. After washing with PBS, the DMEM was replenished, and the cells were visualized and imaged using a confocal microscope (LSM880, Zeiss).

### Protein complex purification

HeLa cells with endogenous FLAG-tagged KCTD7 were harvested and lysed in lysis buffer [20 mM tris-Cl (pH 7.4), 100 mM NaCl, 0.2 mM EDTA, 0.5% NP-40, and 1× protease inhibitor cocktail]. After centrifugation, the WCLs were incubated with anti-FLAG antibody-conjugated M2 agarose beads (Sigma-Aldrich) at 4°C overnight to capture FLAG-tagged KCTD7. The beads were washed five times with lysis buffer and eluted with FLAG peptide. Then, the eluted proteins were separated by SDS–polyacrylamide gel electrophoresis (PAGE) on a gradient gel (Bio-Rad) for Coomassie blue staining. Protein bands were excised from the gel and subjected to sequencing by MS.

### Proximity ligation assays

The in situ interaction between KCTD7 and CLN5 in FLAG-KCTD7 KI HeLa cells was detected using Duolink In situ Detection Reagents (Sigma-Aldrich) according to the manufacturers’ instructions. Briefly, cells were first incubated with anti-FLAG and anti-CLN5 primary antibodies and then with probes consisting of species-specific secondary antibodies conjugated to complementary oligonucleotides, which circularized when the proteins were in close proximity after the addition of hybridization solutions and ligases. Then, polymerase and nucleotides were used to promote rolling circle amplification, which were visualized by red fluorescence and imaged using a confocal microscope (LSM880, Zeiss).

### Deglycosylation

Deglycosylation of WCLs with PNGase F (NEB) was performed according to the manufacturer’s recommendations. Digestion with the enzymes was carried out for 12 hours at 37°C, and then cell lysates were subjected to co-IP assays.

### BiFC assays

The CLN5 and KCTD7 CDSs were inserted into the pBiFC-VN173 and pBiFC-VC155 vectors, respectively. 293T cells were transfected with the indicated BiFC constructs for 24 hours and were then visualized and imaged using a fluorescence microscope (IX73, Olympus). FACS analysis was performed to quantify the percentage of GFP-positive cells.

### In vivo ubiquitination assays

293T cells were transfected with hemagglutinin (HA)–ubiquitin and the indicated plasmids. MG132 (10 μM) was added for 8 hours, and the cells were harvested 36 hours after transfection and lysed in 1% SDS buffer [20 mM tris-Cl (pH 7.4), 0.5 mM EDTA, and 1 mM dithiothreitol] and boiled at 95°C for 10 min. A 10-fold volume of lysis buffer [20 mM tris-Cl (pH 7.4), 100 mM NaCl, 0.2 mM EDTA, 0.5% NP-40, and 1× protease inhibitor cocktail] and anti-FLAG antibody-conjugated M2 agarose beads was added to the product of the previous step. After incubation with shaking at 4°C overnight, the beads were washed four times with BC100 buffer [20 mM tris-Cl (pH 7.9), 100 mM NaCl, 0.2 mM EDTA, 20% glycerol, and 0.2% Triton X-100] and eluted with 3× FLAG peptide for 2 hours at 4°C. Ubiquitinated FLAG-CLN5 was detected by WB using an anti-HA antibody.

To detect the endogenous ubiquitination level of CLN5, parental and KCTD7 KO HeLa cells were transfected with HA-ubiquitin constructs. After 24 hours, the cells were treated with MG132 (10 μM) for 8 hours. Endogenous CLN5 was immunoprecipitated with an anti-CLN5 antibody, and ubiquitinated CLN5 was detected by WB using an anti-HA antibody.

### RUSH cargo sorting assays

The RUSH assay was conducted as previously reported (*36*). HeLa cells were seeded on glass coverslips in 24-well plates and transfected with plasmids containing CLN sequences fused to enhanced GFP (EGFP) and SBP (pStr-KDEL_TPP1-SBP-EGFP, pStr-KDEL_PPT1-SBP-EGFP, and pStr-KDEL_CTSD-SBP-EGFP). At 80% confluence, the cells were incubated with 40 μM d-biotin in cell culture medium for the indicated times. The cells were then washed in PBS, fixed with 4% PFA for 20 min, and imaged with a confocal microscope (LSM880, Zeiss).

### Detection of secreted CLN5

HeLa cells were transfected with different FLAG-CLN5 constructs. Twenty-four hours later, cell pellets were harvested with radioimmunoprecipitation assay buffer [50 mM tris-Cl (pH 7.4), 150 mM NaCl, 1% Triton X-100, 1% sodium deoxycholate, 0.1% SDS, and 1× protease inhibitor cocktail] to detect CLN5 content inside cells (WCLs), while culture medium was incubated with enough anti-FLAG beads to capture secreted CLN5, followed by washing to remove nonspecific binding. Subsequently, the samples were mixed with loading buffer and subjected to SDS-PAGE. The secretion efficiencies of FLAG-CLN5 were calculated according to the sample volumes and WB band intensities of CLN5 in cell lysates and culture media.

### Statistical analysis

Statistical analysis was performed using the GraphPad Prism (GraphPad Software). All data are shown as means ± SD values for experiments performed with at least three replicates. Differences between two groups were analyzed using Student’s *t* test, and differences among multiple groups were analyzed using the one-way or two-way analysis of variance (ANOVA) test.

## Notes

View/request a protocol for this paper from *Bio-protocol*.
