# Supplementary material for: KCTD7 mutations impair the trafficking of lysosomal enzymes through CLN5 accumulation to cause neuronal ceroid lipofuscinoses
Source: Sci Adv. 2022 Aug 3;8(31):eabm5578. doi: 10.1126/sciadv.abm5578 (PMC9348797; doi:10.1126/sciadv.abm5578)
Supplement: Supplementary file 1 — Figs. S1 to S16 Tables S1 to S4 [file sciadv.abm5578_sm.pdf]

Science Advances
AAAS

Supplementary Materials for

KCTD7 mutations impair the trafficking of lysosomal enzymes through CLN5 accumulation to cause neuronal ceroid lipofuscinoses

Yalan Wang *et al.*

Corresponding author: Hongyan Wang, wanghy@fudan.edu.cn; Chenji Wang, chenjiwang@fudan.edu.cn

Sci. Adv. **8**, eabm5578 (2022)
DOI: 10.1126/sciadv.abm5578

The PDF file includes:

Figs. S1 to S16
Tables S1 to S4
Legends for data S1 to S4

Other Supplementary Material for this manuscript includes the following:

Data S1 to S4

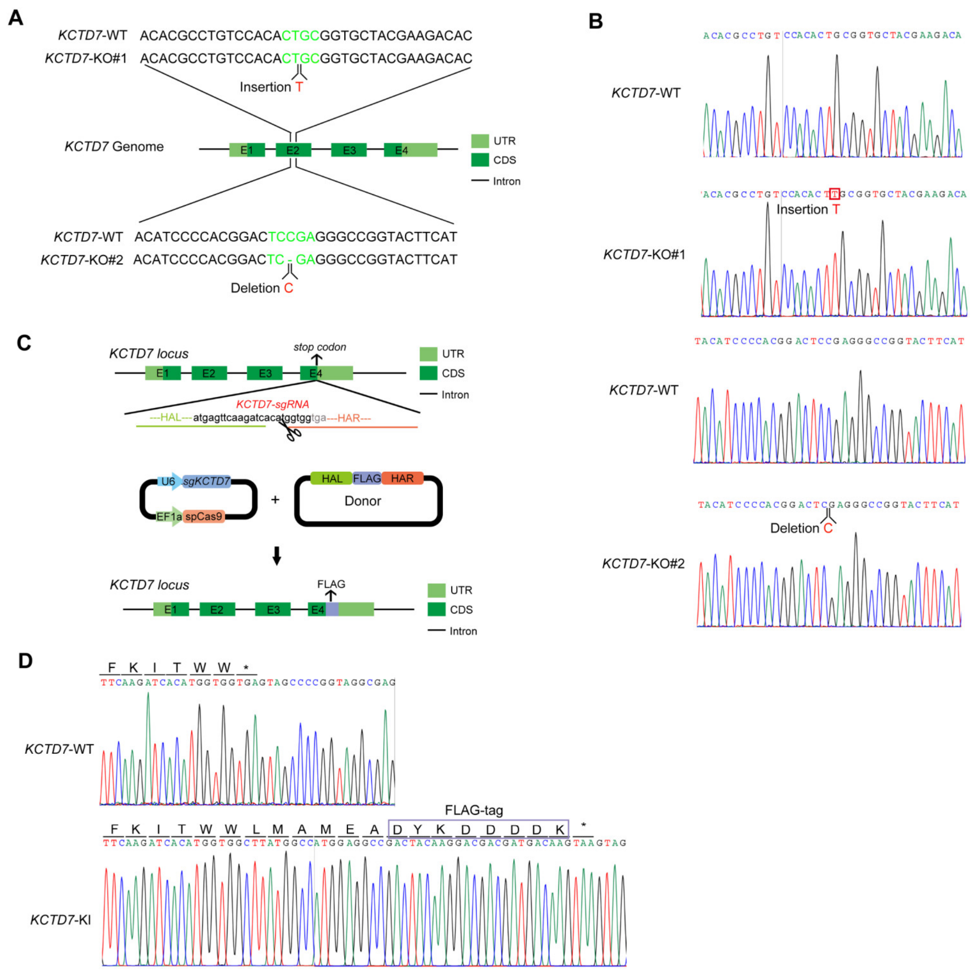

Supplementary Figure. 1 Validation of KCTD7 knockout/knockin in HeLa cells

(A) Schematic diagram of CRISPR/Cas9-mediated knockout of *KCTD7* in HeLa cells.

(B) Sanger sequencing confirming that *KCTD7* gene was edited by frameshift insertion or deletion in HeLa cells.

(C) Schematic diagram of FLAG tag knock-in strategies at the *KCTD7* locus.

(D) Sanger sequencing confirming that FLAG-tag knock-in before the stop codon of the *KCTD7* gene in HeLa cells.

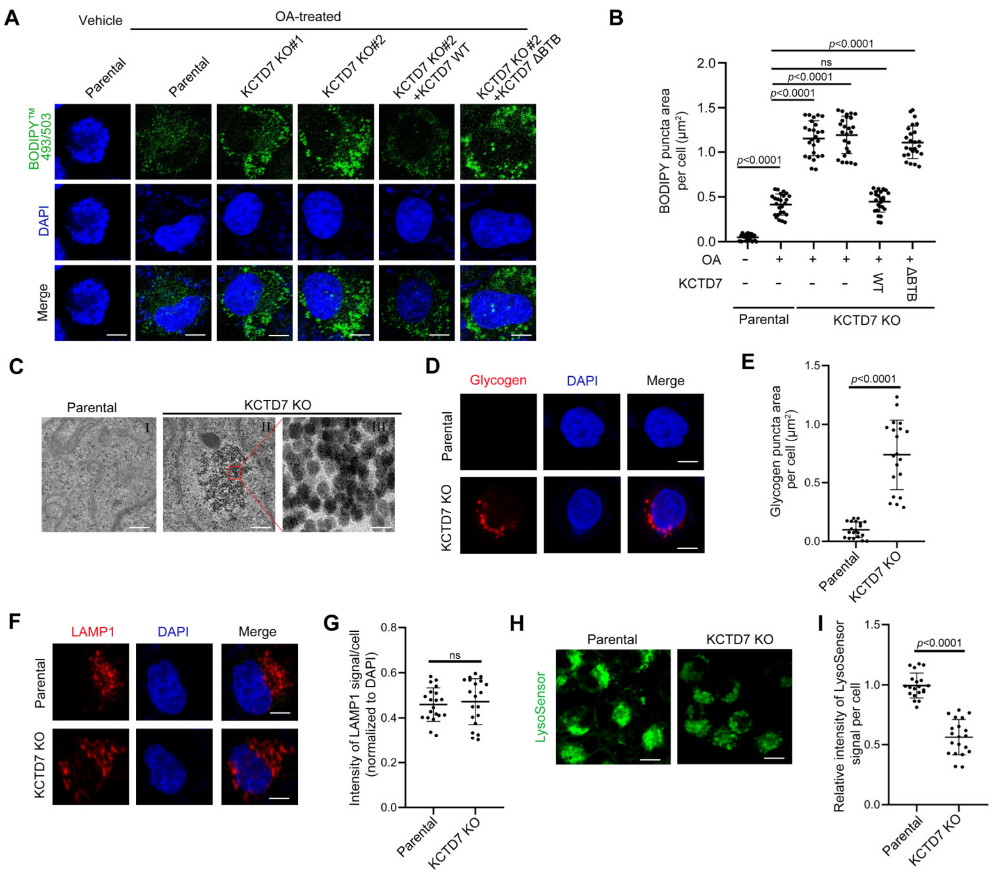

Supplementary Figure. 2 KCTD7 deficiency leads to lysosomal defects in HeLa cells (related to Fig 1)

(A, B) BODIPY 493/503 staining of neural lipid in parental or KCTD7-deficient HeLa cells treated with vehicle or BAS-coupled Oleate acid (OA, 400 μM) for overnight at 33 °C. KCTD7-deficient HeLa cells with DOX-induced FLAG-KCTD7-WT or -ΔBTB mutant are detected in parallel. Scale bar, 10 μm. Quantification of BODIPY 493/503 puncta per cell were shown in (B). Data are presented as mean ± S.D. (n=25).

(C) TEM images showing accumulation of numerous glycogen particles in KCTD7-deficient HeLa cells, but not in parental HeLa cells. Scale bar, 200 nm (I and II), 25 nm (III).

(D, E) Representative IF images of parental or KCTD7-deficient HeLa cells stained with Glycogen (Red), and DAPI. Scale bar, 20 μm. Quantification of Glycogen puncta per cell were shown in (E). Data are presented as mean ± S.D. (n=20).

(F, G) Representative IF images of parental or KCTD7-deficient HeLa cells stained with lysosome marker LAMP1 (Red), and DAPI. Scale bar, 20 μm. Quantification of LAMP1 puncta per cell were shown in (G). Data are presented as mean ± S.D. (n=20).

(H, I) Representative images of parental or KCTD7-deficient HeLa cells stained with

LysoSensor (Green). Scale bar, 20 μm. Quantification of LysoSensor signal intensity per cell were shown in (**I**). Data are presented as mean ± S.D. (n=20).

P values are calculated using One-way ANOVA test (B) or Student's *t* test (E, G and I). ns, not statistically significant.

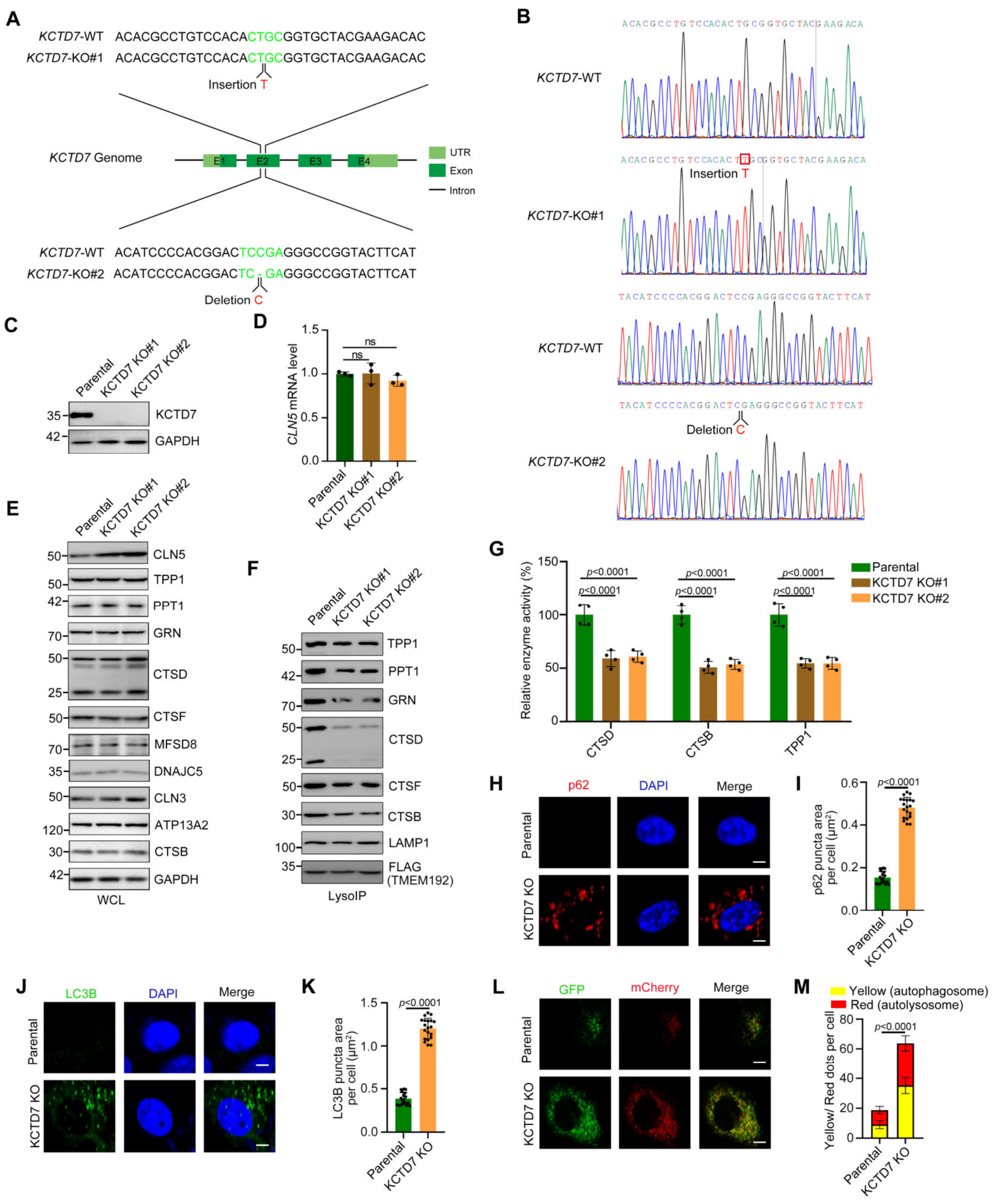

Supplementary Figure. 3 KCTD7 deficiency leads to lysosomal and autophagic defects in U87-MG cells.

(A) Schematic diagram of CRISPR/Cas9-mediated KO of *KCTD7* in U87-MG cells.

(B) Sanger sequencing confirming that *KCTD7* gene was edited by frameshift insertion or deletion in U87-MG cells.

(C) WB analyses of KCTD7 levels in WCLs from parental and KCTD7-deficient U87-MG cells.

(D) RT-qPCR assessment of *CLN5* mRNA levels in parental and KCTD7-deficient U87-MG cells. Data are shown as means ± SD (n=3).

(E) WB analyses of the indicated proteins in WCLs from parental and KCTD7 knockout U87-MG cells.

(F) Lysosomal fractions from parental and KCTD7-deficient U87-MG cells were isolated by LysoIP and then subjected to WB analyses with the indicated antibodies.

(G) Relative enzymatic activities of CTSD, CTSB, and TPP1 in parental and KCTD7-deficient U87-MG cells. Enzymatic activities were measured as relative fluorescence units as compared to parental U87-MG cells.

(H, I) Representative IF images of parental and KCTD7-deficient U87-MG cells stained with p62 and DAPI (H). Scale bar, 10 μm. Quantification of the intensity of p62 signal per cell (I). Data are shown as means ± SD. (n = 20).

(J, K) Representative IF images of parental and KCTD7-deficient U87-MG cells stained with LC3B and DAPI (J). Scale bar, 10 μm. Quantification of the LC3B signal intensity per cell (K). Data are shown as means ± SD. (n = 20).

(L, M) Representative mCherry-GFP-LC3 tandem IF images of parental and KCTD7-deficient U87-MG cells (L). Scale bar, 10 μm. Quantification of the yellow (autophagosome) and red (autolysosome) signal intensity per cell (M). Data are shown as means ± SD. (n = 20).

P values were calculated using the One-way ANOVA test (D and G) and the Student's *t* test (I, K and M). ns, not statistically significant. Band intensity in (E, F) was qualified by ImageJ.

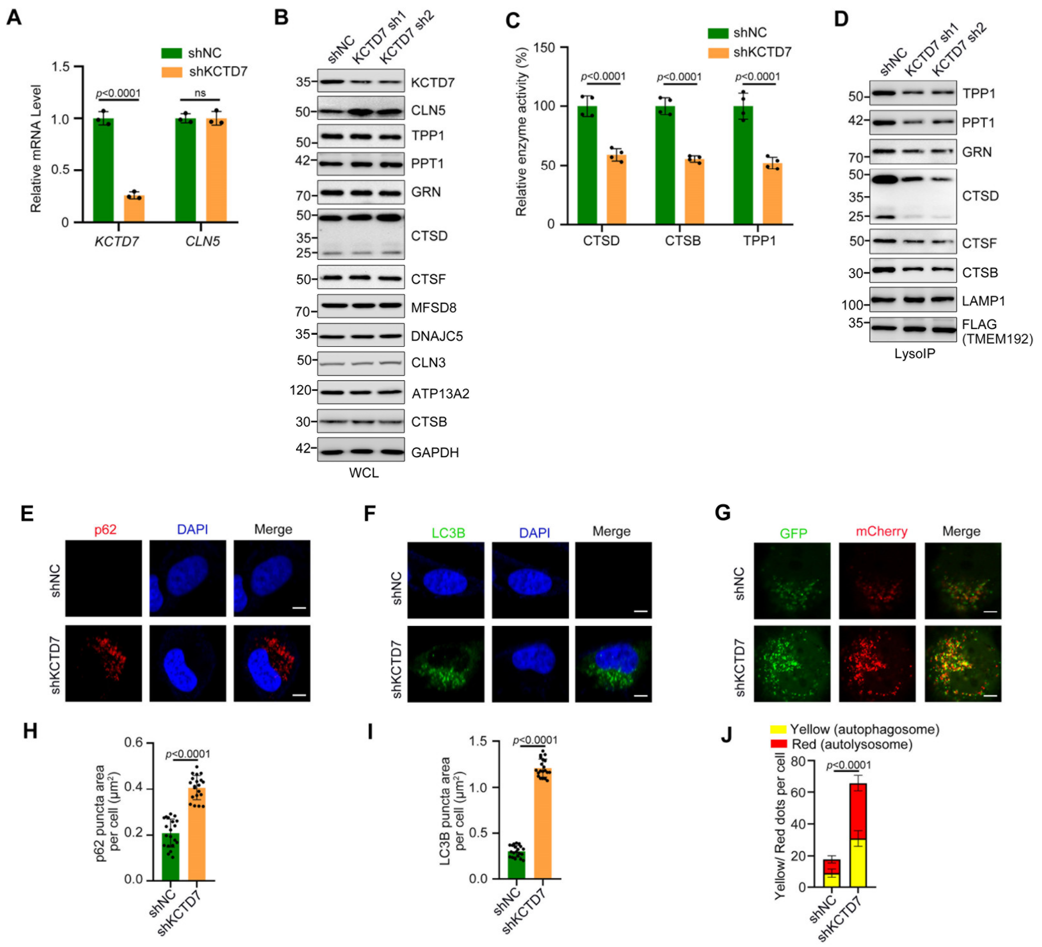

Supplementary Figure. 4 KCTD7 KD leads to lysosomal and autophagic defects in SH-SY5Y cells.

(A) RT-qPCR assessment of *KCTD7* and *CLN5* mRNA levels in SH-SY5Y cells stably overexpressing control shRNA or shRNA targeting KCTD7. Data are shown as means ± SD (n=3).

(B) WB analyses of the indicated proteins in the WCLs from SH-SY5Y cells stably overexpressing control shRNA or shRNAs targeting KCTD7.

(C) Relative enzymatic activities of CTSD, CTSB, and TPP1 in SH-SY5Y cells stably overexpressing control shRNA or shRNA targeting KCTD7. Enzymatic activities were measured as relative fluorescence units as compared to SH-SY5Y cells stably overexpressing control shRNA.

(D) Lysosomal fractions from SH-SY5Y cells stably overexpressing control shRNA or shRNAs targeting KCTD7 were isolated by LysoIP and then subjected to WB analyses with the indicated antibodies.

(E, H) Representative IF images of SH-SY5Y cells stably overexpressing control shRNA or shRNA targeting KCTD7 stained with p62 and DAPI (E). Scale bar, 10 μm. Quantification of the intensity of p62 signal per cell (H). Data are shown as means ± SD. (n = 20).

(F, I) Representative IF images of SH-SY5Y cells stably overexpressing control shRNA or shRNA targeting KCTD7 stained with an LC3B antibody and DAPI (F). Scale bar, 10 μm. Quantification of the LC3B signal intensity per cell (I). Data are shown as means ± SD. (n = 20).

(G, J) Representative mCherry-GFP-LC3 tandem IF images of SH-SY5Y cells stably overexpressing control shRNA or shRNA targeting KCTD7 (G). Scale bar, 10 μm. Quantification of the yellow (autophagosome) and red (autolysosome) signal intensity per cell (J). Data are shown as means ± SD. (n = 20).

P values were calculated using the Student's *t* test (A, C, H, I and J). ns, not statistically significant. Band intensity in (B, D) was qualified by ImageJ.

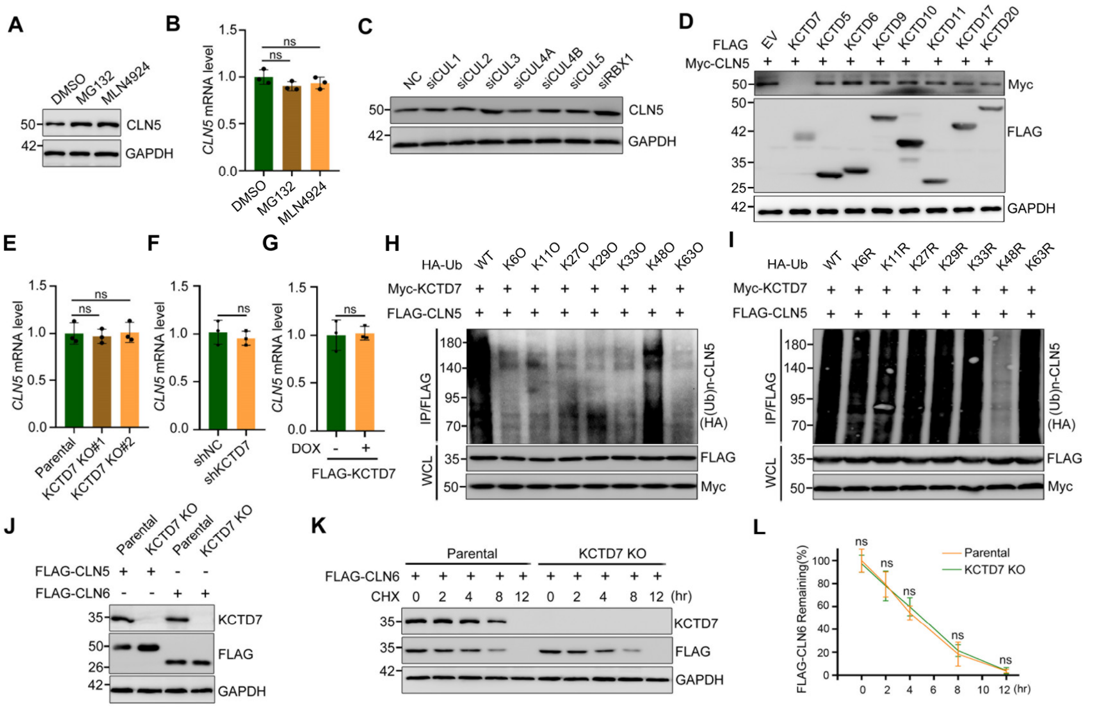

Supplementary Figure. 5 KCTD7 interacts with CLN5 and promotes K48-linked ubiquitination of CLN5.

(A) HeLa cells treated with DMSO, MG132 (10 μM) or MLN4924 (100 nM) for 8 h were harvested and subjected to WB analyses with the indicated antibodies.

(B) RT-qPCR assessment of *CLN5* mRNA levels in HeLa cells treated with DMSO, MG132 (10 μM) or MLN4924 (100 nM) for 8 h.

(C) WB analyses of indicated proteins from HeLa cells transfected with control siRNAs (NC) or siRNAs targeting each Cullin or RBX1.

(D) WB analyses of indicated proteins from 293T cells transfected with the indicated plasmids.

(E-G) RT-qPCR assessment of *CLN5* mRNA levels in parental and KCTD7-deficient HeLa cells (E), in HeLa cells stably overexpressing control shRNA or shRNA targeting KCTD7 (F), in FLAG-KCTD7 Tet-on-inducible HeLa cells treated with DMSO or DOX (10 ng/ml) for 24 h (G).

(H-I) WB analyses of in vivo ubiquitination assays from 293T cells transfected with the indicated plasmids and treated with MG132 (10 μM) for 8 h.

(J) WB analyses of indicated proteins from parental and KCTD7-deficient HeLa cells transfected with the indicated plasmids.

(K, L) WB analyses of indicated proteins from parental or KTCD7-deficient HeLa cells transfected with FLAG-CLN6 and treated with 50 μg/ml CHX and harvested at different time

points (L). At each time point, the intensity of FLAG-CLN6 was normalized to the intensity of GAPDH and then to the value at 0 h.

P values were calculated using the One-way ANOVA test (B and E) or Student's *t* test (F and G). ns, not statistically significant.

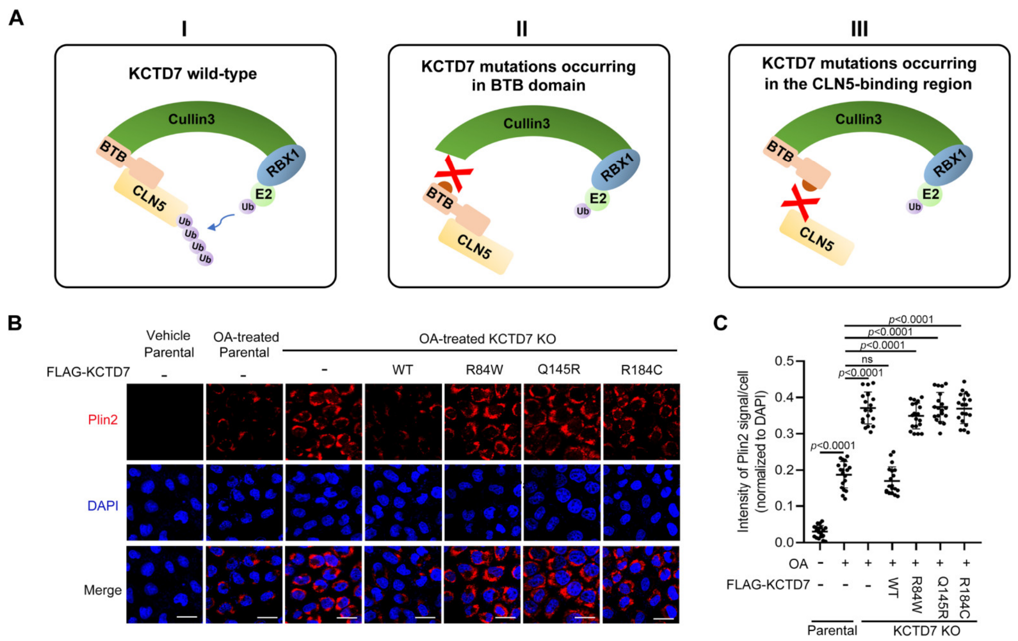

Supplementary Figure. 6 Patient-derived KCTD7 mutants are defective in promoting clearance of Plin2-positive LDs.

(A) A Schematic diagram depicting that KCTD7 mutations occurring in BTB domain or CLN5-binding region impaired CLN5 ubiquitination via different mechanisms.

(B, C) Representative IF images of parental or KCTD7-deficient HeLa cells stably overexpressing EV or Tet-on-inducible FLAG-KCTD7 WT or mutants stained with anti-Plin2 antibody and DAPI. The cells were treated with DMSO or DOX (10 ng/ml) for 3 days and vehicle or OA overnight, followed by being stained with Plin2 and DAPI (B). Scale bar, 50 μm. Quantification of the intensity of Plin2 signal per cell (C). Data are shown as means ± SD (n = 20).

P values were calculated using the One-way ANOVA test (C). ns, not statistically significant.

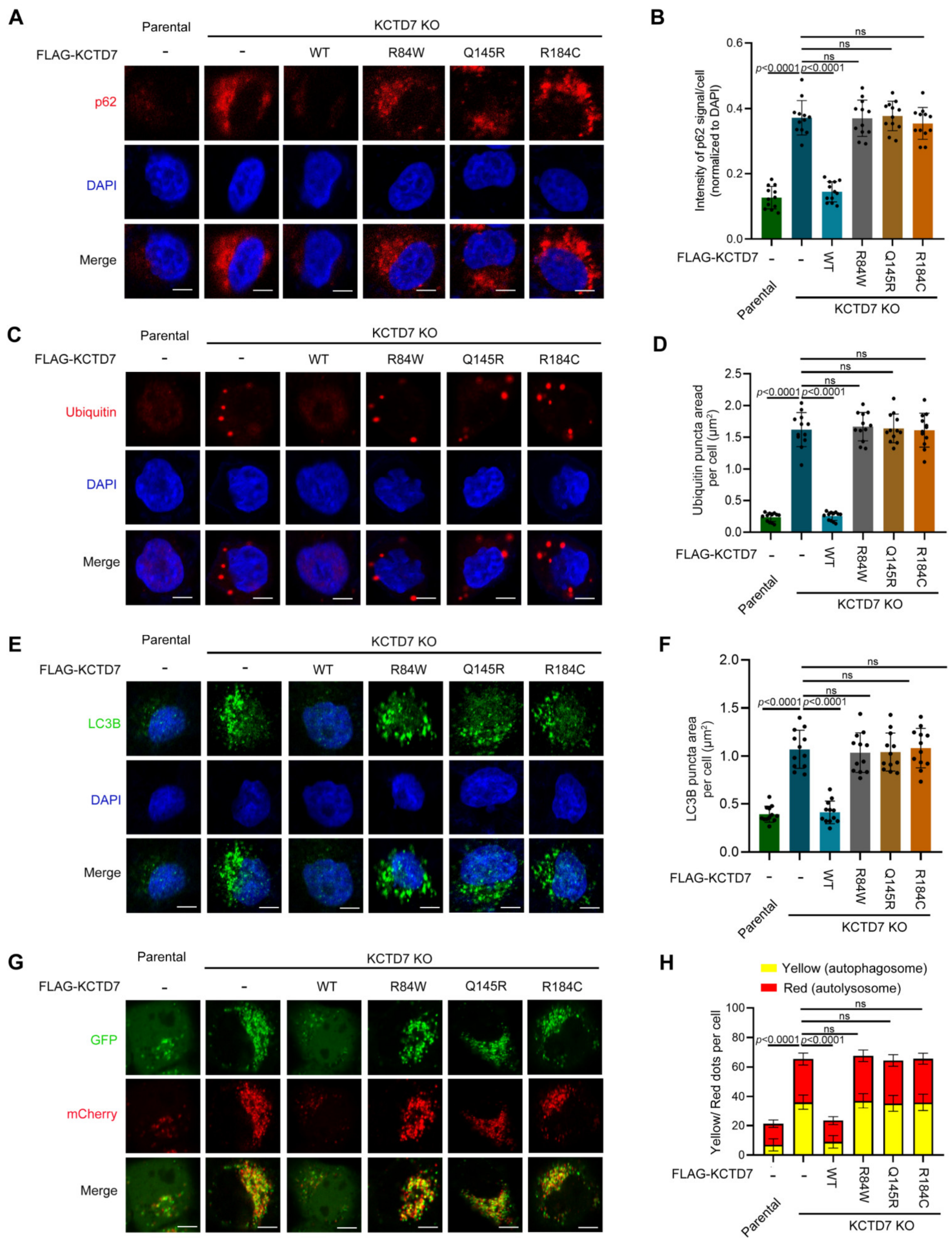

Supplementary Figure. 7 patient-derived KCTD7 mutants fail to reverse autophagic defects caused by KCTD7 deficiency.

(A, B) Representative IF images of parental or KCTD7-deficient HeLa cells stably overexpressing EV or Tet-on-inducible FLAG-KCTD7 WT or mutants. The cells were treated with DMSO or DOX (10 ng/ml) for 3 days and then stained with p62 and DAPI (A). Scale bar, 10 μm. Quantification of the intensity of p62 signal per cell (B). Data are shown as means ± SD (n = 12).

(C, D) Representative IF images of parental or KCTD7-deficient HeLa cells stably overexpressing EV or Tet-on-inducible FLAG-KCTD7 WT or mutants. The cells were treated

with DMSO or DOX (10 ng/ml) for 3 days and then stained with ubiquitin and DAPI (C). Scale bar, 10 μm. Quantification of the intensity of Ubiquitin signal per cell (D). Data are shown as means ± SD. (n = 12).

(E, F) Representative IF images of parental or KCTD7-deficien HeLa cells stably overexpressing EV or Tet-on-inducible FLAG-KCTD7 WT or mutants. The cells were treated with DMSO or DOX (10 ng/ml) for 3 days and then stained with LC3B and DAPI (E). Scale bar, 10μm. Quantification of the intensity of LC3B signal per cell (F). Data are shown as means ± SD. (n = 12).

(G, H) Representative mCherry-GFP-LC3 tandem IF images of parental or KCTD7-deficien HeLa cells stably overexpressing EV or Tet-on-inducible FLAG-KCTD7 WT or mutants. The cells were treated with DMSO or DOX (10 ng/ml) for 3 days (G). Scale bar, 10 μm. Quantification of the intensity of yellow (autophagosome) and red (autolysosome) signal per cell (H). Data are shown as means ± SD (n = 3) in which about 12 cells were quantified.

P values were calculated using the One-way ANOVA test (B, D, F and H). ns, not statistically significant.

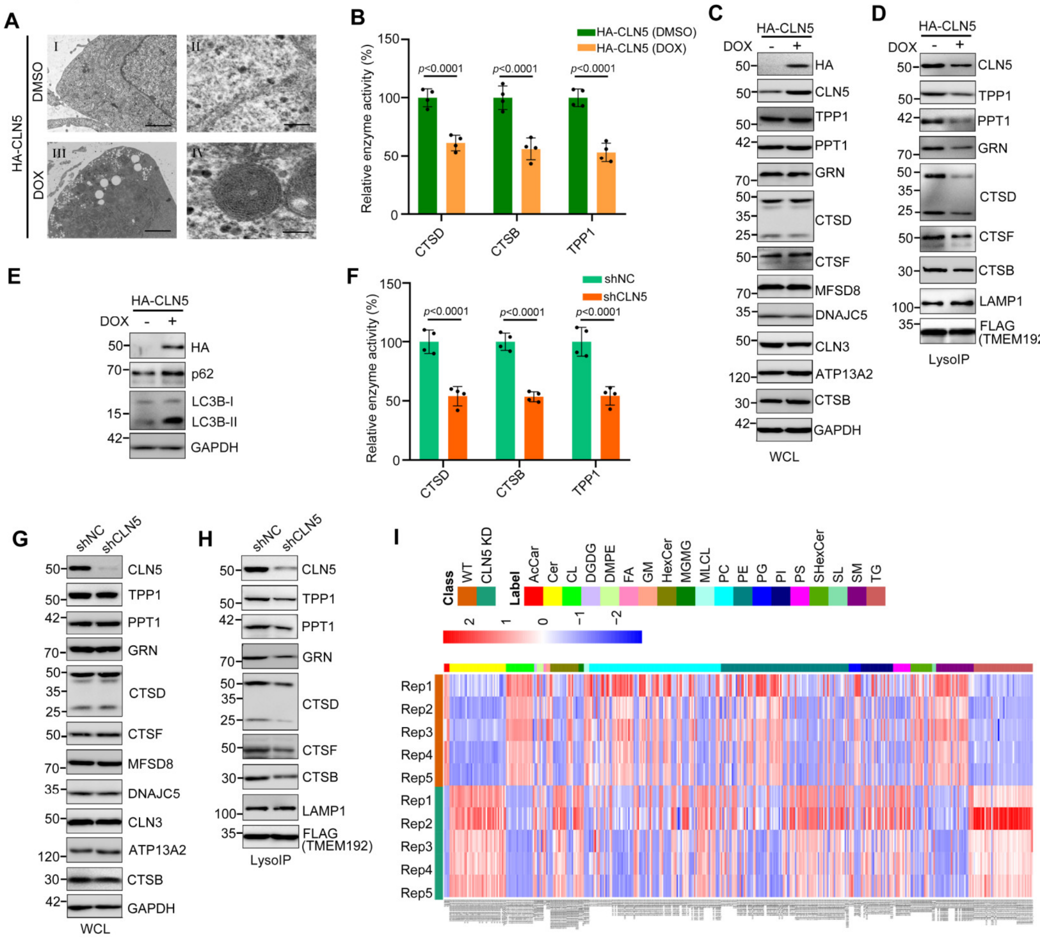

Supplementary Figure. 8 Upregulation and downregulation CLN5 expression both lead to lysosome defects.

(A) TEM images showing lipids (III) and fingerprint-like structure (IV) appeared in HA-CLN5 Tet-on-inducible HeLa cells treated with DOX (10 ng/ml) for 3 days, while these phenotypes were not displayed in DMSO-treated HeLa cells (I, III). Scale bar, 2 μm (I, III); Scale bar, 200 nm (II, IV, V).

(B) Relative enzymatic activities of CTSD, CTSB, and TPP1 in HA-CLN5 Tet-on-inducible HeLa cells treated with DMSO or DOX (10 ng/ml) for 3 days.

(C, D) WB analyses of indicated proteins in the WCLs (C) and lysosomal fractions (D) from HA-CLN5 Tet-on-inducible HeLa cells treated with DMSO or DOX (10 ng/ml) for 3 days.

(E) WB analyses of autophagy markers in WCLs from HA-CLN5 Tet-on-inducible HeLa cells treated with DMSO or DOX (10 ng/ml) for 3 days.

(F) Relative enzymatic activities of CTSD, CTSB, and TPP1 in HA-CLN5 Tet-on-inducible HeLa cells treated with DMSO or DOX (10 ng/ml) for 3 days.

(G, H) WB analyses of indicated proteins in WCLs (G) and lysosomal fractions (H) from HeLa cells stably overexpressing control shRNA or shRNA targeting CLN5.

(I) Lipidomic heatmap showing fold changes in molecular lipid species in HeLa cells stably overexpressing control shRNA or shRNA targeting CLN5. (n = 5).

P values were calculated using the Student's *t* test (B and F) or One-way ANOVA test (K). ns, not statistically significant. Band intensity in (C, D, E, G and H) was qualified by ImageJ.

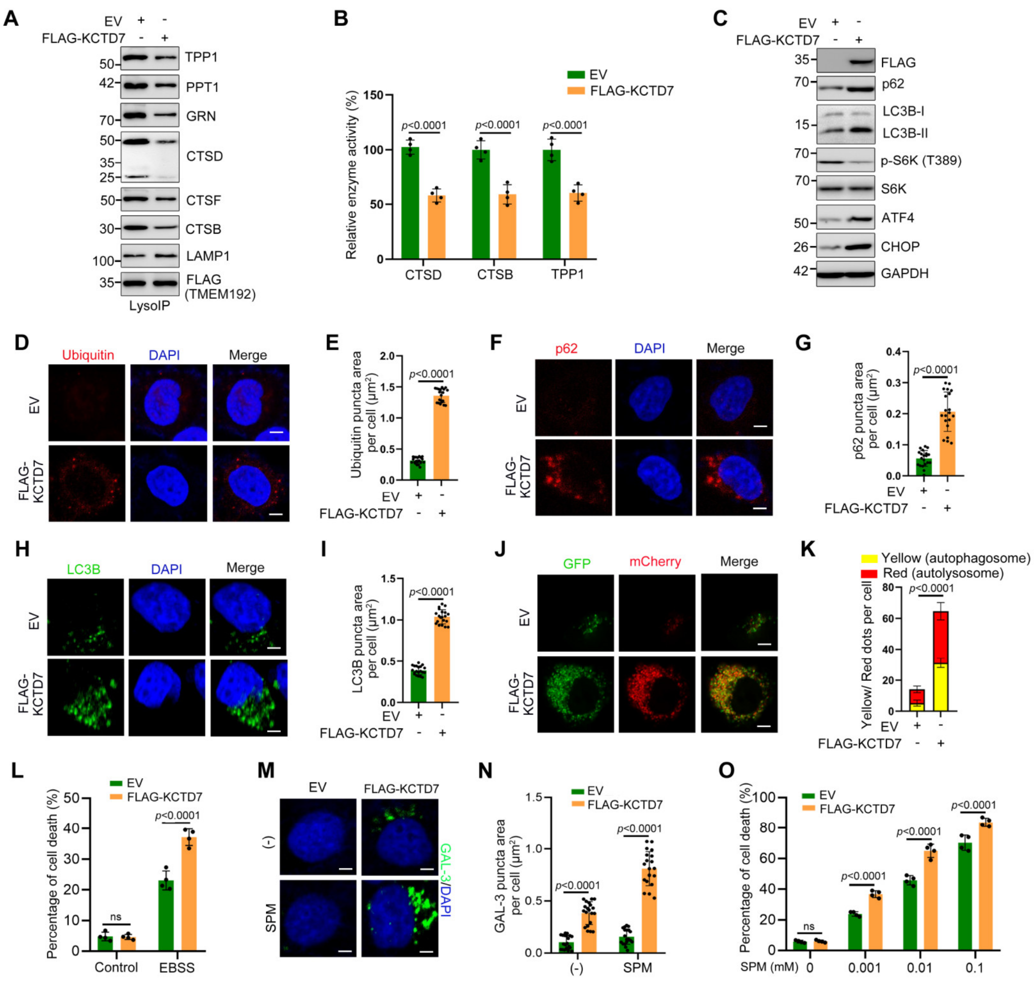

Supplementary Figure. 9 KCTD7 overexpression leads to lysosomal and autophagic defects.

(A) Lysosomal fractions from HeLa cells stably overexpressing EV or KCTD7 were isolated and then subjected to WB analyses with the indicated antibodies.

(B) Relative enzymatic activities of CTSD, CTSB, and TPP1 in HeLa cells stably overexpressing EV or KCTD7. Enzymatic activities were measured as relative fluorescence units as compared to HeLa cells stably overexpressing EV.

(C) WB analysis of WCLs from HeLa cells stably overexpressing EV or KCTD7.

(D-I) Representative IF images of the indicated HeLa cells stained with ubiquitin (D), p62 (F), LC3B (H) and DAPI. Scale bar, 10 μm. Quantification of the intensity of ubiquitin (E), p62 (G), and LC3B (I) signals per cell (D). Data are shown as means ± SD. (n = 12).

(J, K) Representative mCherry-GFP-LC3 tandem immunofluorescence images of HeLa cells stably overexpressing EV or KCTD7 (J). Scale bar, 10 μm. Quantification of the yellow

(autophagosome) and red (autolysosome) signal intensity per cell (K). Data are shown as means ± SD. (n = 20).

(L) Cell death rate (PI staining) of HeLa cells stably overexpressing EV or KCTD7 incubated in control medium (DMEM) or EBSS for 3 h.

(M, N) Representative images of GAL-3 staining in HeLa cells t stably overexpressing EV or KCTD7. Scale bar, 50 μm. Quantification of the area of GAL-3 puncta per cell is shown in (N). Data are presented as mean ± S.D. (n=20).

(O) Cell death rate of HeLa cells stably overexpressing EV or KCTD7 upon treatment with increasing concentrations of SPM for 24 h. Data are presented as mean ± S.D. (n = 3).

P values were calculated using the Student's t test (B, E, G, I, K, L, N and O). ns, not statistically significant. Band intensity in (A, C) was qualified by ImageJ.

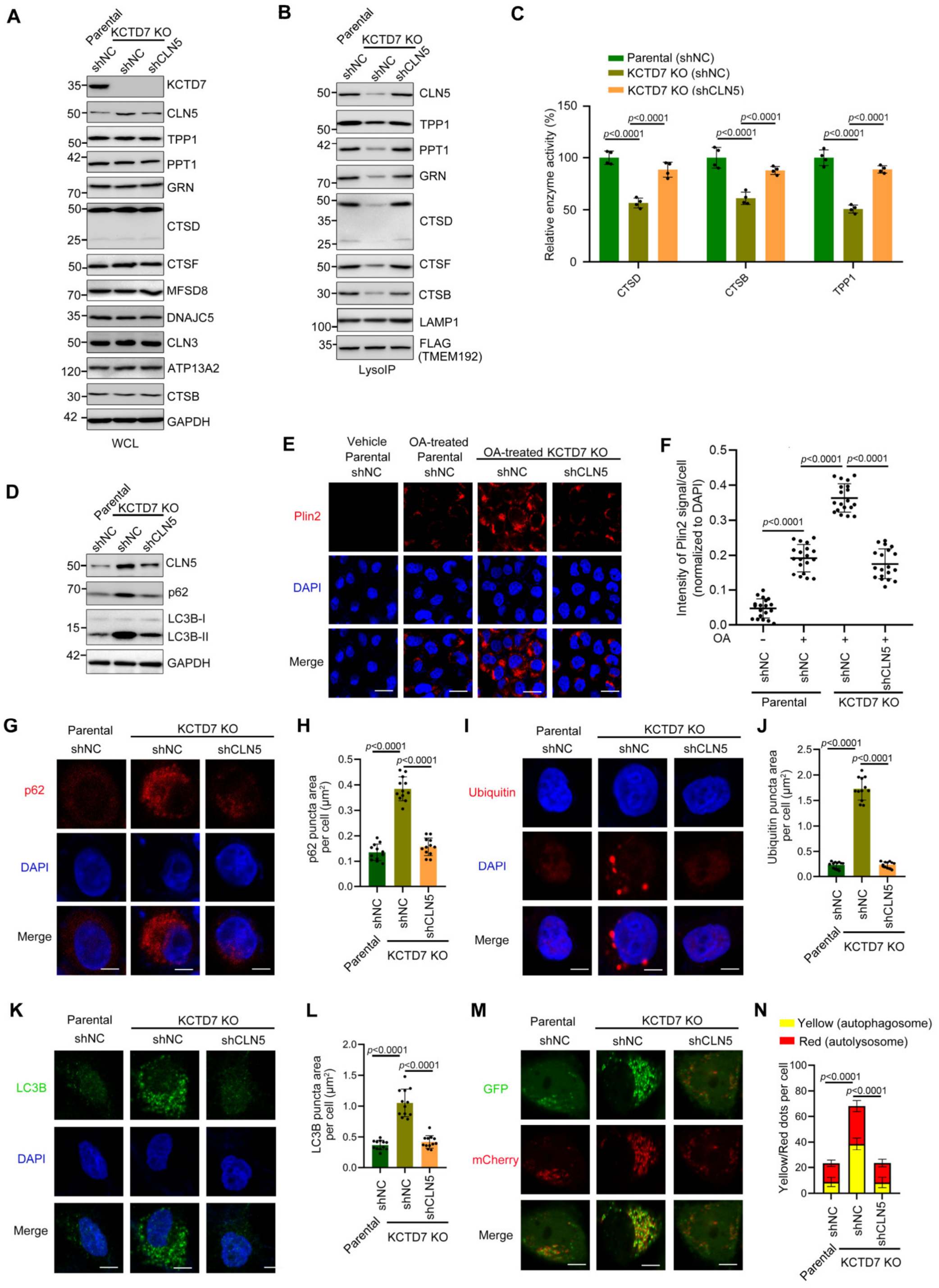

Supplementary Figure. 10 KCTD7 deficiency-caused lysosomal defects can be reversed by CLN5 knockdown.

(A, B) WB of indicated proteins in WCLs (A) and lysosomal fractions (B) from parental or KCTD7-deficient HeLa cells stably overexpressing control shRNA or shRNA targeting CLN5.

(C) Relative enzymatic activities of CTSD, CTSB and TPP1 in parental or KCTD7-deficient HeLa cells stably overexpressing control shRNA or shRNA targeting CLN5.

(D) WB of autophagy markers in WCLs from parental or KCTD7-deficient HeLa cells stably overexpressing control shRNA or shRNA targeting CLN5.

(E, F) Representative IF images of parental or KCTD7-deficient HeLa cells stably overexpressing control shRNA or shRNA targeting CLN5 (E). The cells were treated with vehicle or OA and then stained with Plin2 and DAPI. Scale bar, 50 μm. Quantification of the intensity of Plin2 signal per cell (F). Data are shown as means ± SD. (n = 20).

(G-L) Representative IF images of the indicated HeLa cells stained with p62 (G), ubiquitin (I), LC3B (K) and DAPI. Scale bar, 10 μm. Quantification of the intensity of p62 (H), ubiquitin (J), and LC3B (L) signals per cell (D). Data are shown as means ± SD. (n = 12).

(M, N) Representative mCherry-GFP-LC3 tandem IF images of parental or KCTD7-deficient HeLa cells stably overexpressing control shRNA or shRNA targeting CLN5 (M). Scale bar, 10μm. Quantification of the intensity of Yellow (autophagosome) and Red (autolysosome) signal per cell (N). Data are shown as means ± SD (n = 12).

P values were calculated using One-way the ANOVA test (B, D, F, H and J). ns, not statistically significant. Band intensity in (A, B, D) was qualified by ImageJ.

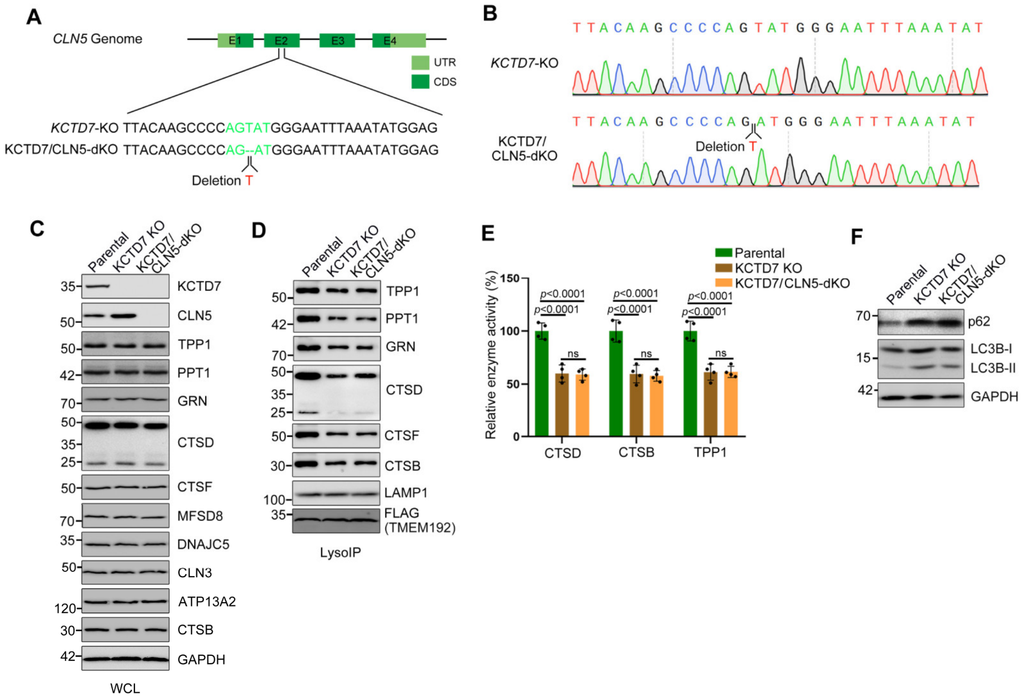

Supplementary Figure. 11 KCTD7/CLN5 double-KO HeLa cells show similar lysosomal and autophagic defects as KCTD7 KO cells.

(A) Schematic diagram of CRISPR/Cas9-mediated KO of *CLN5* in *KCTD7*-KO HeLa cells.

(B) Sanger sequencing confirming that *CLN5* gene was edited by frameshift deletion in *KCTD7* KO HeLa cells.

(C) WB analyses of the indicated proteins in WCLs from parental, KCTD7 KO and KCTD7/CLN5 dKO HeLa cells.

(D) Lysosomal fractions from parental, KCTD7 KO and KCTD7/CLN5 dKO HeLa cells were isolated by LysoIP and then subjected to WB analyses with the indicated antibodies.

(E) Relative enzymatic activities of CTSD, CTSB, and TPP1 in parental, KCTD7 KO and KCTD7/CLN5 dKO HeLa cells. Enzymatic activities were measured as relative fluorescence units as compared to parental HeLa cells.

(F) WB analyses of the indicated proteins in WCLs from parental, KCTD7 KO, and KCTD7/CLN5 dKO HeLa cells.

P values were calculated using the One-way ANOVA test (E). Band intensity in (C, D and F) was qualified by ImageJ.

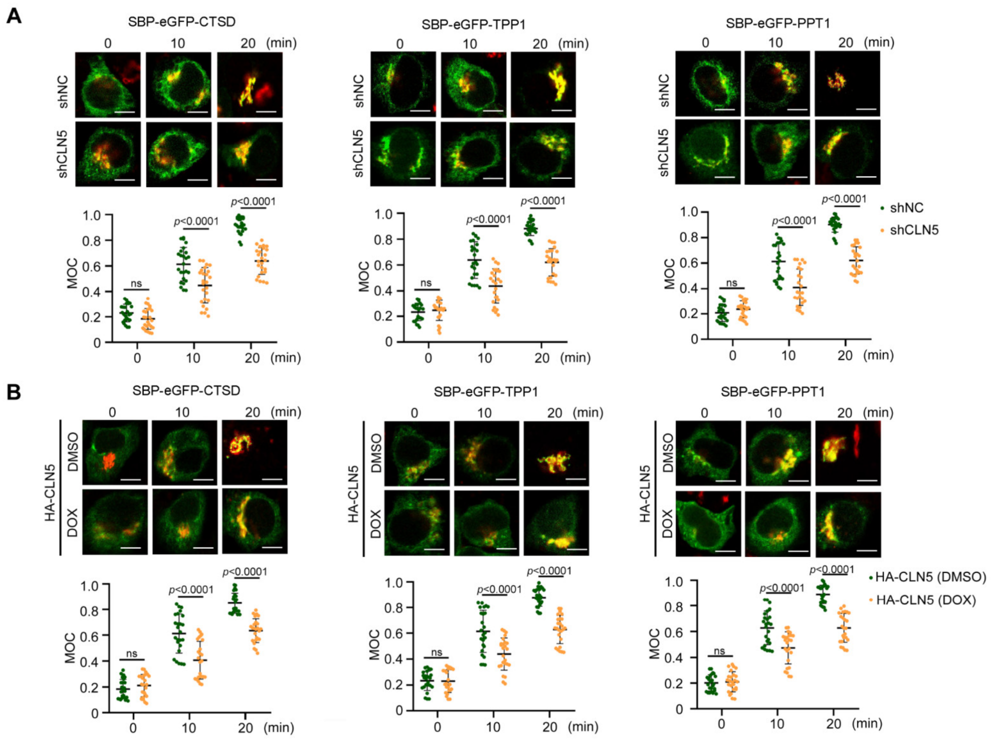

Supplementary Figure. 12 CLN5 KD or overexpression leads to impaired ER-to-Golgi trafficking of lysosomal enzymes.

(A) Confocal colocalization analysis of SBP-GFP-fused lysosomal enzymes (CTSD, TPP1, PPT1; green) with Golgi marker GM130 in HeLa cells stably overexpressing control shRNA or shRNA targeting CLN5 by RUSH. Representative IF images are shown in the upper panel (Scale bar, 10 μm), and the colocalization degree between the test proteins and GM130 was quantified by Manders' overlap coefficient (MOC) and is shown in the lower panel. Data are presented as mean ± S.D. (n = 20). *P* values are calculated using Two-way ANOVA test. ns, not statistically significant.

(B) Confocal colocalization analysis of SBP-GFP-fused lysosomal enzymes with Golgi marker GM130 in HA-CLN5 Tet-on-inducible HeLa cells treated with DMSO or DOX (10 ng/ml) for 3 days by RUSH. Representative IF images are shown in upper panel (Scale bar, 10 μm), and quantifications of the colocalization degree between test proteins and GM130 are measured by MOC and shown in lower panel. Data are presented as mean ± S.D. (n=20).

P values were calculated using the Student's *t* test (A and B). ns, not statistically significant.

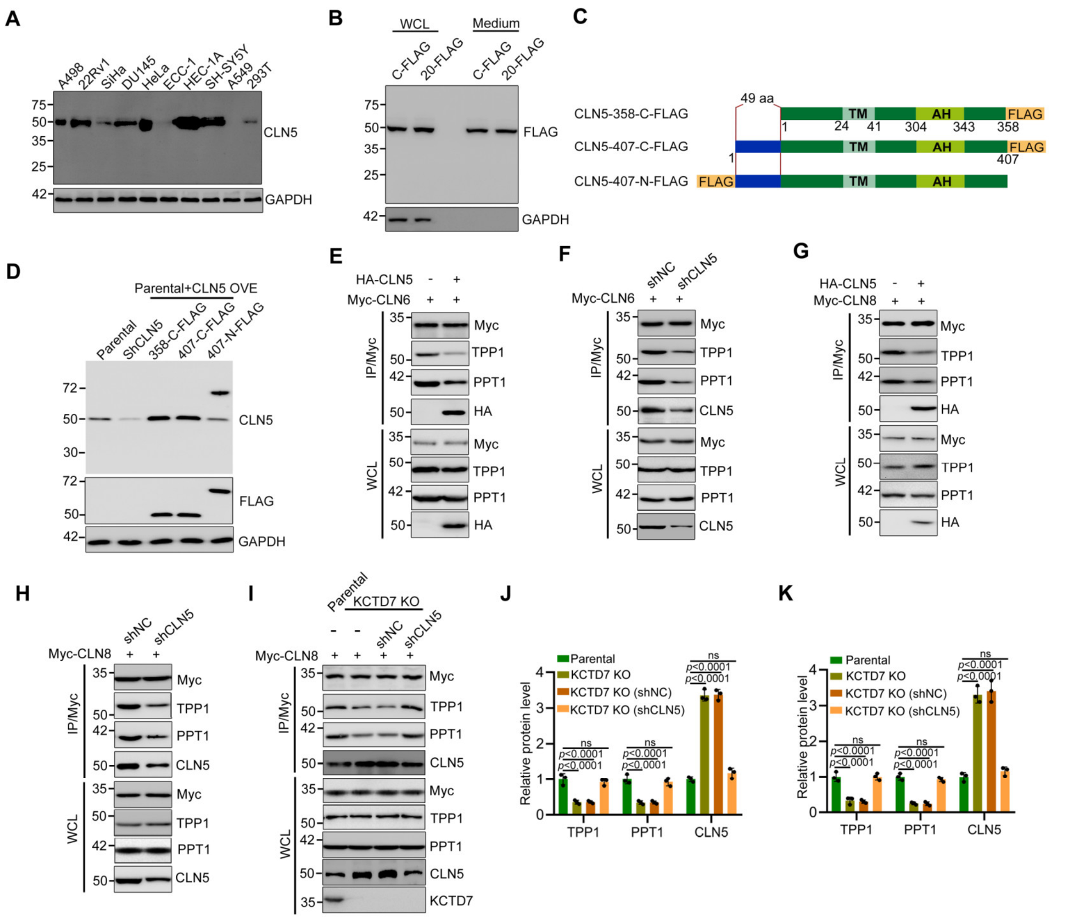

Supplementary Figure. 13 KCTD7 deficiency disrupts lysosomal enzyme trafficking from ER to Golgi through CLN5 accumulation (related to Figure. 6).

(A) WB analyses of endogenous CLN5 in the WCLs from multiple human cell lines.

(B) WB analyses of the indicated proteins in the WCLs and culture media from 293T cells transfected with indicated plasmids. 1/20 volume of WCLs and 1/2 volume of FLAG-CLN5 enriched immunoprecipitates from culture medium were loaded.

(C) The schematic diagram indicating constructs of CLN5 proteins tagged by N-terminal, or, C-terminal FLAG.

(D) WB analyses of the indicated proteins in the WCLs from 293T cells transfected indicated plasmids.

(E) HeLa cells were transfected with indicated plasmids. The WCLs were prepared and subjected to co-IP assay.

(F) HeLa cells stably overexpressing control shRNA or shRNA targeting CLN5 were transfected with Myc-CLN6 for 24 h. The WCLs were prepared and subjected to co-IP with anti-Myc

antibody. The WCLs and immunoprecipitates were prepared for WB analyses with the indicated antibodies.

(G) HeLa cells were transfected with indicated plasmids. The WCLs were prepared and subjected to co-IP assay.

(H) The indicated HeLa cells were transfected with Myc-CLN8 for another 24 h. The WCLs were prepared and subjected to co-IP assay.

(I) The indicated HeLa cells with/without CLN5 KD were transfected with EV, or Myc-CLN8 for 24 h. Then the WCLs were prepared and subjected to co-IP assay.

(J) Quantification of the intensity of indicated protein precipitated by Myc-CLN6 in parental and KCTD7 KO cells with/without CLN5 KD in Fig. 6F.

(K) Quantification of the intensity of indicated protein precipitated by Myc-CLN8 in parental and KCTD7 KO cells with/without CLN5 KD in (I).

P values were calculated using the One-way ANOVA test (J and K). ns, not statistically significant. Band intensity in (E, F, G, H, I) was qualified by ImageJ.

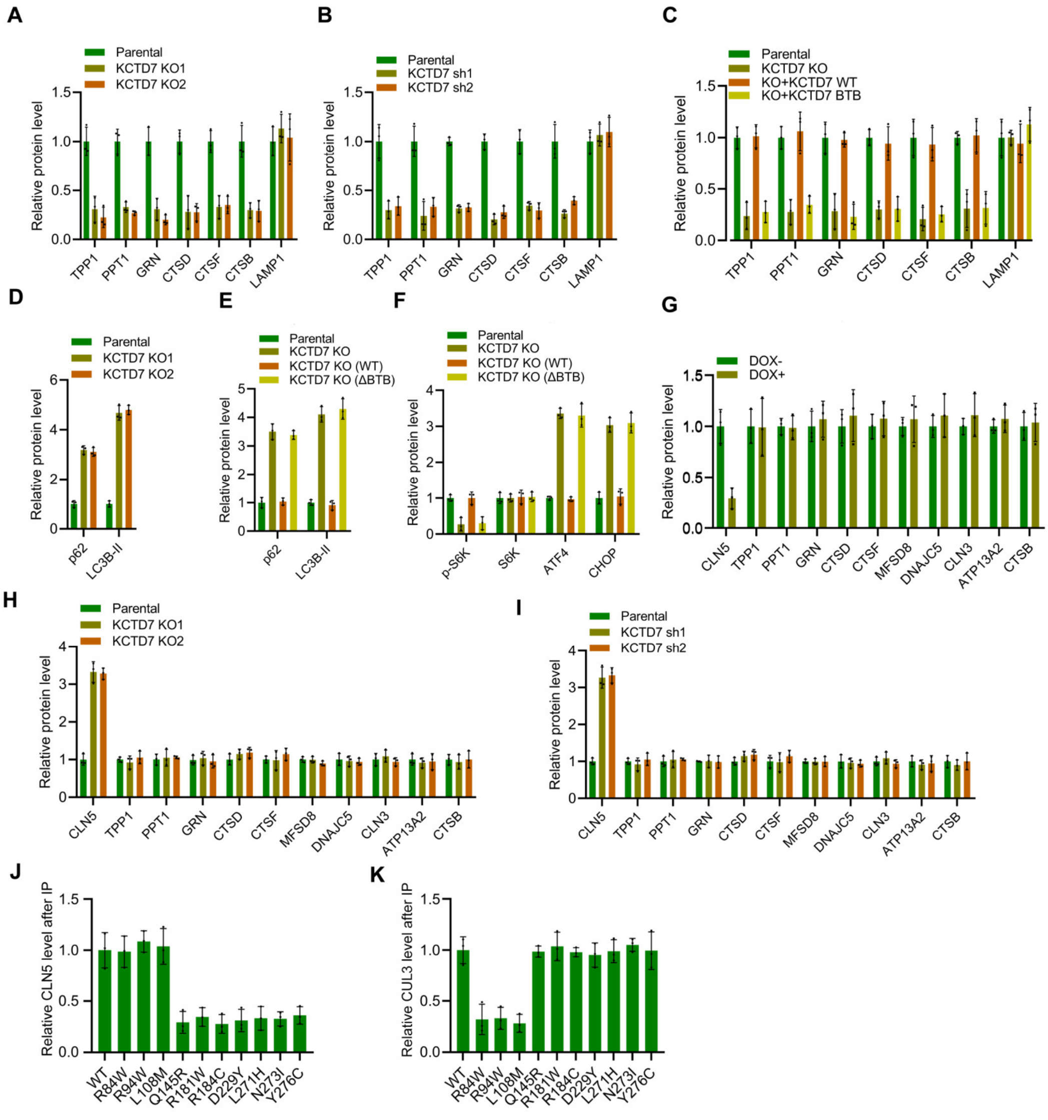

Supplementary Figure. 14 Protein Quantification

(A) Quantification of the intensity of indicated protein in Fig. 1G. The Western blot detection for this experiment was performed in triplicates. The intensity of each band was normalized to the intensity of FLAG-TMEM192.

(B) Quantification of the intensity of indicated protein in Fig. 1H. The Western blot detection for this experiment was performed in triplicates. The intensity of each band was normalized to the intensity of FLAG-TMEM192.

(C) Quantification of the intensity of indicated protein in Fig. 1I. The Western blot detection for this experiment was performed in triplicates. The intensity of each band was normalized to the intensity of FLAG-TMEM192.

(D) Quantification of the intensity of indicated protein in Fig. 2I. The Western blot detection for this experiment was performed in triplicates. The intensity of each band was normalized to the intensity of GAPDH.

(E) Quantification of the intensity of indicated protein in Fig. 2J. The Western blot detection for this experiment was performed in triplicates. The intensity of each band was normalized to the intensity of GAPDH.

(F) Quantification of the intensity of indicated protein in Fig. 2N. The Western blot detection for this experiment was performed in triplicates. The intensity of each band was normalized to the intensity of GAPDH.

(G) Quantification of the intensity of indicated protein in Fig. 4E. The Western blot detection for this experiment was performed in triplicates. The intensity of each band was normalized to the intensity of GAPDH.

(H) Quantification of the intensity of indicated protein in Fig. 4F. The Western blot detection for this experiment was performed in triplicates. The intensity of each band was normalized to the intensity of GAPDH.

(I) Quantification of the intensity of indicated protein in Fig. 4G. The Western blot detection for this experiment was performed in triplicates. The intensity of each band was normalized to the intensity of GAPDH.

(J, K) Quantification of the intensity of CLN5 (J) and CUL3 (K) after IP in Figure. 5E. The Western blot detection for this experiment was performed in triplicates.

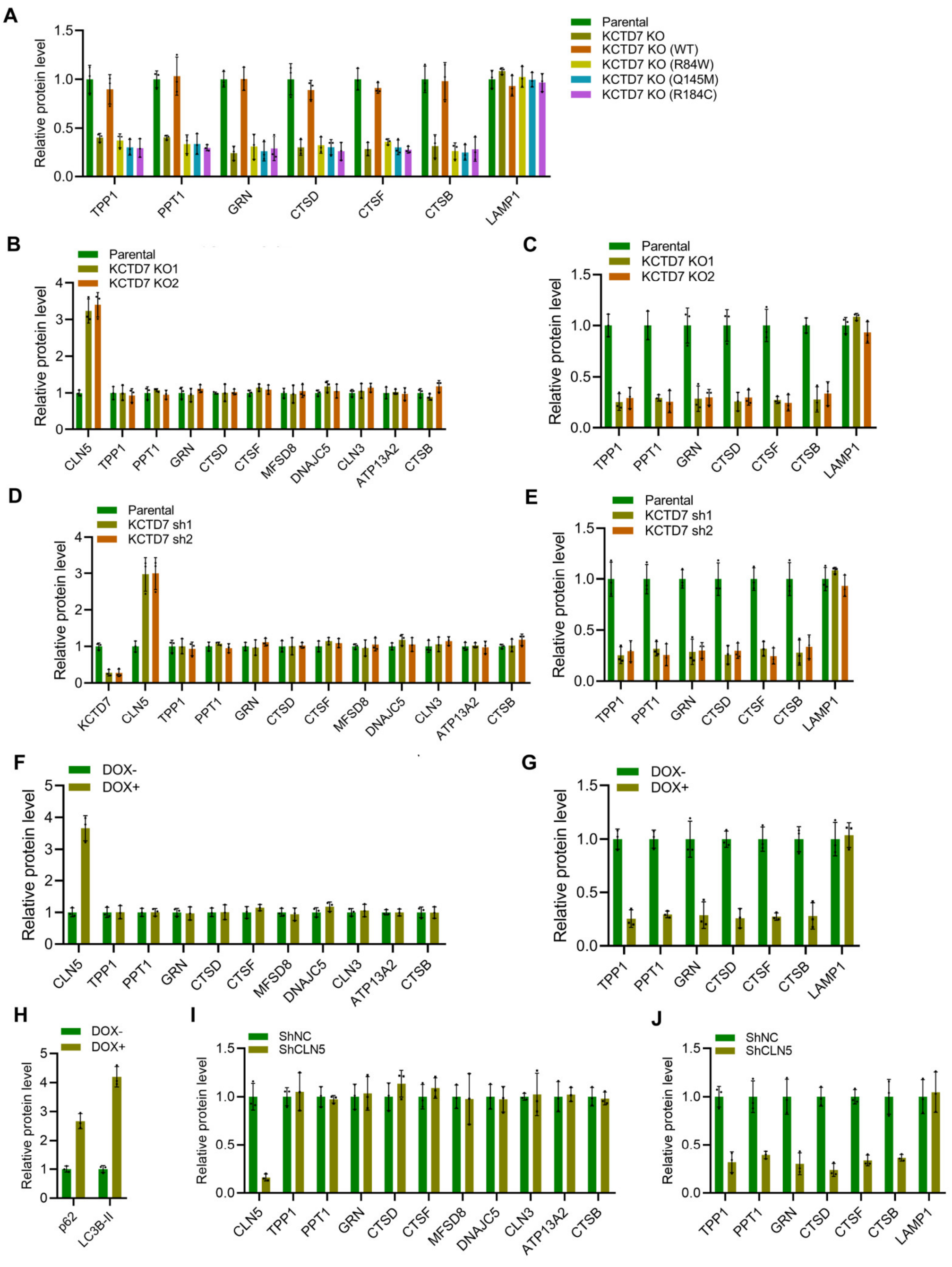

Supplementary Figure. 15 Protein Quantification

(A) Quantification of the intensity of indicated protein in Fig. 5G. The Western blot detection for this experiment was performed in triplicates. The intensity of each band was normalized to the intensity of FLAG-TMEM192.

(B) Quantification of the intensity of indicated protein in Supplementary Fig. 3E. The Western blot detection for this experiment was performed in triplicates. The intensity of each band was normalized to the intensity of GADPH.

(C) Quantification of the intensity of indicated protein in Supplementary Fig. 3F. The Western blot detection for this experiment was performed in triplicates. The intensity of each band was normalized to the intensity of FLAG-TMEM192.

(D) Quantification of the intensity of indicated protein in Supplementary Fig. 4B. The Western blot detection for this experiment was performed in triplicates. The intensity of each band was normalized to the intensity of FLAG-TMEM192.

(E) Quantification of the intensity of indicated protein in Supplementary Fig. 4D. The Western blot detection for this experiment was performed in triplicates. The intensity of each band was normalized to the intensity of FLAG-TMEM192.

(F) Quantification of the intensity of indicated protein in Supplementary Fig. 8C. The Western blot detection for this experiment was performed in triplicates. The intensity of each band was normalized to the intensity of GAPDH.

(G) Quantification of the intensity of indicated protein in Supplementary Fig. 8D. The Western blot detection for this experiment was performed in triplicates. The intensity of each band was normalized to the intensity of FLAG-TMEM192.

(H) Quantification of the intensity of indicated protein in Supplementary Fig. 8E. The Western blot detection for this experiment was performed in triplicates. The intensity of each band was normalized to the intensity of GAPDH.

(I) Quantification of the intensity of indicated protein in Supplementary Fig. 8G. The Western blot detection for this experiment was performed in triplicates. The intensity of each band was normalized to the intensity of GAPDH.

(J) Quantification of the intensity of indicated protein in Supplementary Fig. 8H. The Western blot detection for this experiment was performed in triplicates. The intensity of each band was normalized to the intensity of FLAG-TMEM192.

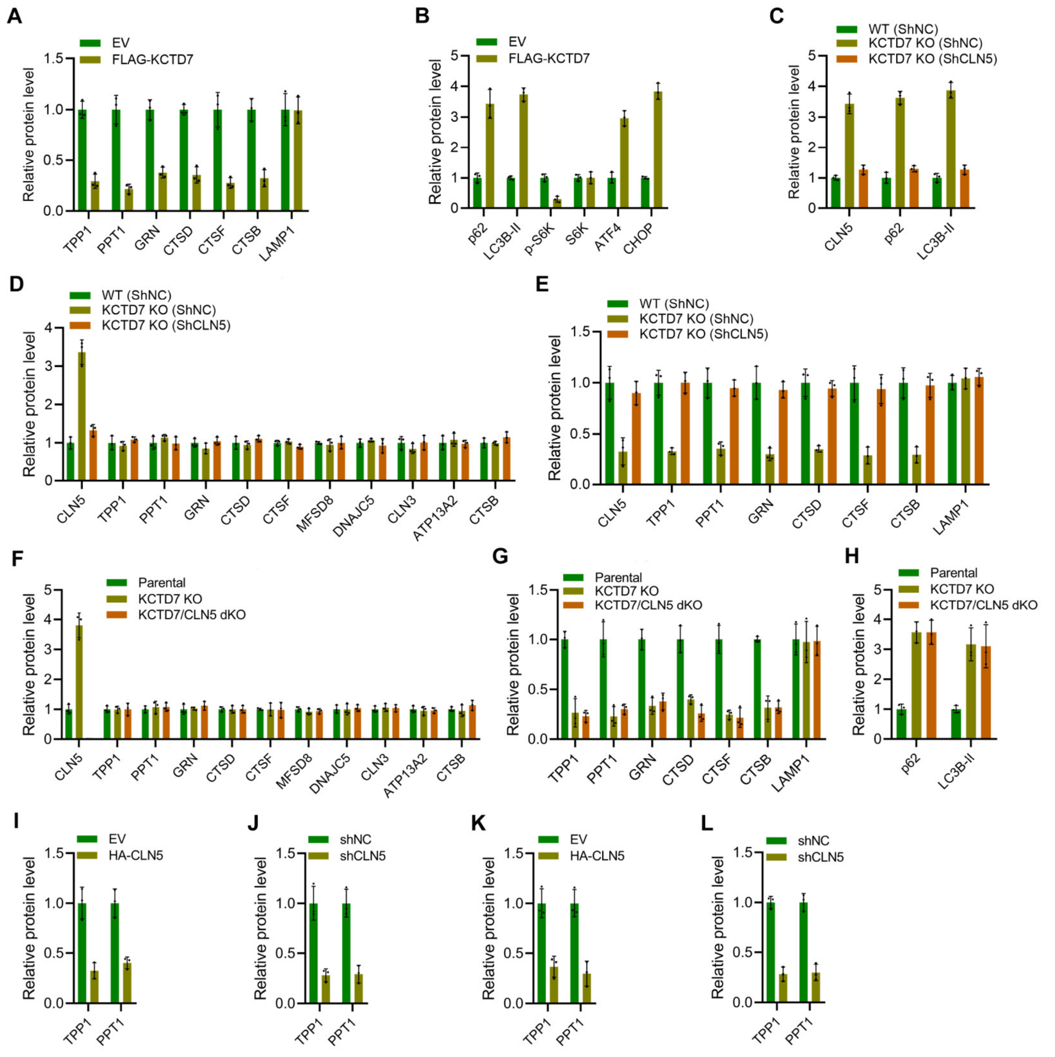

Supplementary Figure. 16 Protein Quantification

(A) Quantification of the intensity of indicated protein in Supplementary Fig. 9A. The Western blot detection for this experiment was performed in triplicates. The intensity of each band was normalized to the intensity of FLAG-TMEM192.

(B) Quantification of the intensity of indicated protein in Supplementary Fig. 9C. The Western blot detection for this experiment was performed in triplicates. The intensity of each band was normalized to the intensity of GADPH.

(C) Quantification of the intensity of indicated protein in Supplementary Fig. 10D. The Western blot detection for this experiment was performed in triplicates. The intensity of each band was normalized to the intensity of GAPDH.

(D) Quantification of the intensity of indicated protein in Supplementary Fig. 10A. The Western blot detection for this experiment was performed in triplicates. The intensity of each band was normalized to the intensity of GAPDH.

(E) Quantification of the intensity of indicated protein in Supplementary Fig. 10B. The Western blot detection for this experiment was performed in triplicates. The intensity of each band was normalized to the intensity of FLAG-TMEM192.

(F) Quantification of the intensity of indicated protein in Supplementary Fig. 11C. The Western blot detection for this experiment was performed in triplicates. The intensity of each band was normalized to the intensity of GAPDH.

(G) Quantification of the intensity of indicated protein in Supplementary Fig. 11D. The Western blot detection for this experiment was performed in triplicates. The intensity of each band was normalized to the intensity of FLAG-TMEM192.

(H) Quantification of the intensity of indicated protein in Supplementary Fig. 11F. The Western blot detection for this experiment was performed in triplicates. The intensity of each band was normalized to the intensity of GAPDH.

(I) Quantification of the intensity of indicated protein after IP in Supplementary Fig. 13E. The Western blot detection for this experiment was performed in triplicates.

(J) Quantification of the intensity of indicated protein after IP in Supplementary Fig. 13F. The Western blot detection for this experiment was performed in triplicates.

(K) Quantification of the intensity of indicated protein after IP in Supplementary Fig. 13G. The Western blot detection for this experiment was performed in triplicates.

(L) Quantification of the intensity of indicated protein after IP in Supplementary Fig. 13H. The Western blot detection for this experiment was performed in triplicates.

Supplementary Table. 1 Genotype and phenotype of cases carrying KCTD7 mutations from published studies.

Mutations		Onset (Months)	Symptoms	Reference (PMID)
Allele 1	Allele 2			
T64A	R211X	23	myoclonus, movement disorder, dyskinesia, dystonia, choreoath	30295347
R70W	R84Q	20	myoclonus, seizures, ataxia	30295347
R84W	D106fs	5	myoclonus, seizures, movement disorder, dyskinesia	22638565
R94W	R94W	10	myoclonus, seizures, ataxia, dyskinesia	22606975<br>22693283
R99X	R99X	18	myoclonus, seizures, ataxia	17455289
G105E	G114E	16	myoclonus, seizures, movement disorder, ataxia, dyskinesia	30295347
L108M	L108M	13-18	myoclonus, seizures, movement disorder, ataxia, choreoath	22693283<br>22612257<br>27293772
R112C	R112C	22	myoclonus, seizures, movement disorder, developmental delay, ataxia, dystonia,	30295347
D115Y	N273I	18	myoclonus, seizures, ataxia	22693283
R121L	W235S	18	myoclonus, seizures, movement disorder, developmental delay, dyskinesia	30295347
R153H	R153H	15	myoclonus, seizures, movement disorder, ataxia, choreoath	30295347
R177H	A178V	24	myoclonus, seizures, developmental delay, dyskinesia	30295347
V179fs	V179fs	10-19	myoclonus movement disorder, ataxia	30295347
R181W	D229Y	24	myoclonus, seizures, movement disorder, developmental delay, dyskinesia	30295347
R184C	R184C	8-9	myoclonus, seizures,	22748208
I199fs	I199fs	9-18	myoclonus, seizures, ataxia	22693283
P205L	V259E	16	myoclonus, seizures, developmental delay, ataxia, dyskinesia, dystonia, choreoath	30295347
D229Y	L271H	15	myoclonus, movement disorder, developmental delay, ataxia, dystonia, choreoath	30295347
F232fs	F232fs	6-9	myoclonus, seizures, movement disorder, ataxia	27742667
N273I	N273I	18	myoclonus, seizures	22693283
Y276C	Y276C	15-24	myoclonus, seizures, developmental delay, ataxia	25060828
W289X	W289X	18-36	myoclonus, seizures, movement disorder, developmental delay, ataxia	22693283
T64A	S113P	21	cognitive and motor deterioration, ataxia, epileptic paroxysms, cerebellar subatrophy	32412666
G58R	G58R	24	myoclonus; developmental delay	31972370
A178V	A178V	13	myoclonic, ataxia; developmental regression; loss of speech	31972370
L147P	A174T	21-24	myoclonus, dystonia, movement disorder	31247399
Q145R	R211X	24	myoclonic seizures, tonic-clonic seizures, and neurological deterioration	30825425

Supplementary Table. 2 Oligonucleotides used for genome editing, RT-qPCR, and gene knockdown.

Name	Forward Primer (5'-3')	Reverse Primer (5'-3')
**Genome editing**		
KCTD7_KO_sg1	ccgggggcccgatggcatagtac	aacgtactatgccatcgggcccc
KCTD7_KO_sg2	ccggaaggtgcgccaagcgtttc	aacgaaacgcttggcgcaccttc
KCTD7_KO_PCR	gggattgaagatggagcagc	ggctgtctttgtctcctttgg
KCTD7_KI_sg	ccgatgagttcaagatcacatgg	aacccatgtgatcttgaactcat
KCTD7_KI_PCR	gatgcccatcacccccctat	agctatcagcctcaaggaaag
CLN5_KO_sg1	ccgtggcgctgctttggctcgcg	aaccgcgagccaaagcagcgcca
CLN5_KO_sg1	ccgtcgattacaagccccagtat	aacatactggggcttgtaatcga
**RT-qPCR**		
KCTD7-RT	gagggccggtacttcatcg	catgagtcccagaaacgcttg
CLN5-RT	ctgatggcgcaggaggtag	ttttggacggaagtcaaagcg
GAPDH_RT	gaaggtgaaggtcggagtc	gaagatggtgatgggatttc
**gene knockdown**		
KCTD7 Sh1	gcttcgcgccgtagtctta	
KCTD7 Sh2	ggtgctacgaagacaccatgt	
ShCLN5	gcaactatatcaggaaacatg	
siRBX1#1	gaagcgcuuugaagugaaa	
siRBX1#2	Gggauauugugguugauaa	
siRBX1#3	ggaaccacauuauggaucu	
siRBX1#4	cauagaaugucaagcuaac	
siCUL1#1	caacgaagaguucagguuu	
siCUL1#2	cgaggaagaccgcaaacua	
siCUL1#3	agacagugcuugauguuca	
siCUL1#4	cauagaagacaaagacgua	
siCUL2#1	ggaagugcaugguaaauuu	
siCUL2#2	cauccaaguucauauacua	
siCUL2#3	gcagaaagacacaccacaa	
siCUL2#4	ugguuuaccucauaugauu	
siCUL3#1	gagaagatgtactaaattc	
siCUL3#2	cgacagaaaacatgagata	
siCUL3#3	gaagtagacgacgacaga	
siCUL3#4	gagatcaagttgtacgtta	
siCUL4A#1	gcacagauccuuccguuua	
siCUL4A#2	gaacagcgaucguaaucaa	
siCUL4A#3	gcauguggauucaaaguua	

siCUL4A#4	gcgaguacaucaagacuuu
siCUL4B#1	uaaauaaccuccuugauga
siCUL4B#2	cagaagucauuaauugcua
siCUL4B#3	cggaaagagugcaucugua
siCUL4B#4	gcuauuggccgacauaugu
siCUL5#1	gacacgacgucuuauauua
siCUL5#2	gcaaauagaguggcuaaua
siCUL5#3	uaaacaagcuugcuagaau
siCUL5#4	cgucuaaucuguuaaagaa

Supplementary Table. 3 Lists of antibodies.

Antibodies	Supplier	Catalog	Host	Dilution/Concentration
Anti-p62	MBL	M162-A59	Mouse	1:1000 (WB), 1:200 (IF)
Anti-LC3B	CST	11930	Rabbit	1:1000 (WB), 1:200 (IF)
Anti-Multi Ubiquitin Mouse	MBL	D058-3	Mouse	1:1000 (WB), 1:200 (IF)
Anti-LAMP1 (H4A3)	Santacruz	sc-20011	Mouse	1:1000 (WB), 1:200 (IF)
Anti- SCMAS	Abcam	ab181243	Rabbit	1:200 (IF)
Anti-Plin2	PTG	15294-1-AP	Rabbit	1:200 (IF)
Anti-Glycogen	Hitoshi ASHIDA Ph.D	ESG1A9mAb	Rabbit	1:200 (IF)
Anti-GAL3	PTG	14979-1-AP	Rabbit	1:200 (IF)
Anti-CLN5	Abcam	ab170899	Rabbit	1:1000 (WB), 1:200 (IF)
Anti-PPT1	GeneTex	GTX110677	Rabbit	1:500 (WB)
Anti-TPP1	PTG	12479-1-AP	Rabbit	1:500 (WB)
Anti-CLN3	Abclonal	A1931	Rabbit	1:500 (WB)
Anti-DNAJC5	Abclonal	A10489	Rabbit	1:500 (WB)
Anti-MFSD8	Millipore	HPA044802	Rabbit	1:500 (WB)
Anti-CTSD	Abcam	ab75852	Rabbit	1:1000 (WB)
Anti-GRN	PTG	18410-1-AP	Rabbit	1:1000 (WB)
Anti-CTSB	Abcam	ab214428	Rabbit	1:1000 (WB)
Anti-CTSF	Abcam	A10469	Rabbit	1:1000 (WB)
Anti-ATP13A2	A13083	A13083	Rabbit	1:500 (WB)
Anti-KCTD7	Abclonal	Custom	Rabbit	1:500 (WB)
Anti-CUL3	CST	10450	Rabbit	1:1000 (WB)
Anti-p70 S6K	CST	2708S	Rabbit	1:1000 (WB)
Anti-p-pS6K (T389)	CST	9205S	Rabbit	1:1000 (WB)
Anti-ATF4	CST	11815S	Rabbit	1:1000 (WB)
Anti-CHOP	Santa Cruz	sc-7351	Mouse	1:500 (WB)

Anti-FLAG	CST	14793	Rabbit	1:1000 (WB), 1:200 (IF)
Anti-Myc	MBL	M047-A59	Mouse	1:200 (IF)
Anti-Myc	CTS	2272	Rabbit	1:1000 (WB)
Anti-Myc	Abmart	M20002	Mouse	1:1000 (WB)
Anti-HA	MBL	M180-7	Mouse	1:1000 (WB)
Anti-HA	CTS	3724	Rabbit	1:1000 (WB)
Anti-GAPDH	PTG	AC033	Mouse	1:10000 (WB)
Goat anti-mouse IgG, Alexa Fluor 488	Thermo Scientific	A-11017	Goat	1:500 (IF)
Donkey anti-Mouse IgG, Alexa Fluor Plus 594	Thermo Scientific	A32744	Donkey	1:500 (IF)
Goat anti-Rabbit IgG, Alexa Fluor 488	Thermo Scientific	A-11008	Goat	1:500 (IF)
Goat anti-Rabbit IgG, Alexa Fluor 594	Thermo Scientific	A-11012	Goat	1:500 (IF)
Goat Anti-Rabbit IgG-HRP	Abmart	M21002L	Goat	1:10000 (WB)
Goat Anti-Mouse IgG HRP	Abmart	M21001L	Goat	1:10000 (WB)
Goat Anti-Mouse IgG HRP (light chain)	Abmart	M21004S	Goat	1:10000 (WB)

Supplementary Table. 4 Cell cultures, chemicals, and Kits

Reagent	Source	Identifier
DMEM	Gibco	Cat#11960044
Fetal Bovine Serum	Gibco	Cat# 10099
Penicillin-Streptomycin	Invitrogen	Cat#15070063
EBSS	Gibco	Cat#24010043
MG132	Selleck	Cat#S2619
MLN4924	Selleck	Cat#S7109
Puromycin	Sigma	Cat#P8833
Cycloheximide	APExBIO	S7418
Lipofectamine 2000	Thermo Fisher	Cat#11668027
Lipofectamine 3000	Thermo Fisher	Cat#L3000008
FLAG peptide	ChinaPeptides	Cat#04010006736
Phanta Max Super-Fidelity DNA Polymerase	Vazyme Biotech	Cat#P505
KOD-Plus-Mutagenesis Kit	TOYOBO	Cat#SMK-101
Duolink ® In Situ PLA® Probe Anti-Mouse MINUS kit	Sigma	Cat# SLBZ8548
Duolink ® In Situ PLA® Probe Anti-Rabbit MINUS kit	Sigma	Cat# SLBZ9865
ClonExpress II One Step Cloning Kit	Vazyme Biotech	Cat#C112
Anti-FLAG M2 Magnetic Beads	Sigma	Cat#M8823
Anti-FLAGM2 Affinity Gel	Sigma	Cat#A2220

Hoechst 33342	Thermo Scientific	Cat#62249
DAPI	Sigma	Cat#D9542
Oleat Acid	Sangon Biotech	Cat#A502071-0250
BODIPY 493/503	Thermo Scientific	Cat#D3922
Doxycycline	Selleck	Cat#S5159
d-Biotin	Sangon Biotech	Cat#A600078-0001
TRIzon	Wbiotech	Cat#CW0580S
HiScript III 1st Strand cDNA Synthesis Kit	Vazyme	Cat#R312-01
ChamQ SYBR qPCR Master Mix	Vazyme Biotech	Cat#Q311
LysoSensor	YEASEN	Cat#40767ES50
Minute™ ER Enrichment Kit	Invent	Cat#ER-036
Propidium Iodide	Abcam	Cat# ab14083
SPM	Selleck	S3948
PNGase F	NEB	P0704S

Other Supplementary Material for this manuscript includes the following:

Data S1 to S4

Supplementary Data. 1 The data of differential lipid metabolites between parental and KCTD7-deficient HeLa cells.

Supplementary Data. 2 The data of differential lipid metabolites between parental and CLN5-deficient HeLa cells.

Supplementary Data. 3 MS results of FLAG-tagged endogenous KCTD7 complex.

Supplementary Data. 4 MS results of overexpressed FLAG-CLN5.
